# Exotic-looking Neotropical Tischeriidae (Lepidoptera) and their host plants

**DOI:** 10.3897/zookeys.970.54801

**Published:** 2020-09-21

**Authors:** Jonas R. Stonis, Arūnas Diškus, Andrius Remeikis, M. Alma Solis, Liliana Katinas

**Affiliations:** 1 Institute of Ecology, Nature Research Centre, Akademijos St. 2, Vilnius LT-08412, Lithuania; 2 Systematic Entomology Laboratory, Beltsville Agriculture Research Center, Agriculture Research Center, U.S. Department of Agriculture, National Museum of Natural History, Smithsonian Institution, Washington, D.C., 20013-7012, USA; 3 División Plantas Vasculares, Museo de La Plata, Paseo del Bosque s/n, 1900 La Plata, Argentina

**Keywords:** *
Astrotischeria
*, distribution range, leaf mines, new species, *
Paratischeria
*

## Abstract

Seven new species of Tischeriidae are described from the Neotropics: *Astrotischeria jociui* Diškus & Stonis, **sp. nov.** (feeding on *Wissadula excelsior* (Cav.) C. Presl., Malvaceae), *A. atlantica* Diškus & Stonis, **sp. nov.** (feeding on *Baccharis spicata* (Lam.) Baill., Asteraceae), *A. cornuata* Diškus & Stonis, **sp. nov.** (host plant unknown), *Paratischeria guarani* Diškus & Stonis, **sp. nov.** (feeding on *Elephantopus mollis* Kunth, Asteraceae), *P. mesoamericana* Diškus & Stonis, **sp. nov.** (feeding on *Montanoa hibiscifolia* Benth., Asteraceae), *P. suprafasciata* Diškus & Stonis, **sp. nov.** (feeding on *Allophyllus edulis* (A. St.-Hil., A. Juss. & Cambess.) Hieron. ex Niederl., Sapindaceae), and *P. braziliensis* Diškus & Stonis, **sp. nov.** (host plant unknown). Additionally, an updated distribution map of *Paratischeria neotropicana* (Diškus & Stonis, 2015), which currently has the broadest distribution range among the Neotropical Tischeriidae is provided along with new host-plant data, a list of all recorded host plants in the Neotropics, and a brief discussion on trophic relationships of Tischeriidae. It is hypothesized that host-plant distribution ranges can provide clues to potential distribution ranges of these specialized, monophagous or oligophagous, leaf miners. All new taxa are illustrated with photographs of the adults, their genitalia, and, if available, leaf mines.

## Introduction

Biodiversity inventories provide knowledge about nature and are of utmost importance to understand the complicated mechanisms of the global biota. It is also essential for providing tools for prompt measures in the preservation of biodiversity in the face of a biodiversity crisis and climate change. Along with other organisms, trumpet moths (Tischerioidea: Tischeriidae) can provide data in support of hypotheses about the earlier genesis of the Earth’s biota. They also been used as an express tool for monitoring biodiversity, rapid assessment of biodiversity plots of critical value, and determining priority areas from the environmental point of view in the tropical America (Stonis, unpublished). However, tischeriids are not well-known or very common in museum holdings worldwide and are probably among the least studied lepidopteran groups in tropical and subtropical areas worldwide, including the Neotropics (Stonis et al. 2019b, 2020b). Nevertheless, they are a distinct family from the oldest (monotrysian) lineages of extant Lepidoptera (see Regier 2015 for a phylogenetic discussion) and very peculiar morphologically (Stonis et al. 2020b). Larvae of Tischeriidae are leaf miners of wild and cultivated plants; they mine inside green tissues during all instars and produce irregular, usually blotch-like leaf mines (Figs 5–9, 11–14, 17–21, 25–31, 35–37), but sometimes these are slender and sinuous or have another shape. Pupation occurs inside the leaf mine, often in a round, silken-lined nidus (Figs 31, 131, 137). Adults of trumpet moths (Figs 38–49) are very small, 5–10 mm in wingspan, with the 3^rd^ antennal segment greatly enlarged (see Stonis et al. 2017: Fig. 67). Males possess long antennal sensillae trichodea, which usually exceed the width of the flagellum by more than 4.5–10 times and have strongly recurved, sometimes thickened bases (see Stonis et al. 2017: Fig. 67). In the male genitalia, the phallus is strongly narrowed and usually bifurcated or with spines at its apex. In the female genitalia, the ovipositor lobes are covered with short, dark, thickened peg-like setae; along the stout anterior and posterior apophyses, there are three additional pairs of unique, rod-like or plate-like projections collectively referred as prela. For detailed morphological and biological characterization of this group of tiny leaf miners, we refer to Braun (1972), Puplesis and Diškus (2003), Stonis et al. (2018a); and for generic diagnostics we recommend Puplesis and Diškus (2003), Stonis et al. (2017, 2018a), and Xu et al. (2017). The phylogenetic position of Tischeriidae was discussed by Regier et al. (2015).

The study of the Tischeriidae fauna in the Neotropics began with descriptions of two species from the Caribbean (Walsingham 1897), one from southwestern Mexico (Walsingham 1914), one species from Guyana, and three species from Ecuador and Peru (Meyrick 1915c). After a long break, Bourquin (1962) added one more species from Argentina. The study of Neotropical Tischeriidae has become more resolute and dynamic with targeted, additional fieldwork during the last two decades (Puplesis and Diškus 2003; Landry and Roque-Albelo 2004; Stonis and Diškus 2007, 2008; Stonis et al. 2008, 2016, 2017, 2018a, 2019b, 2019c, 2019d, 2020a, 2020b; Navickaitė et al. 2011; Diškus et al. 2014; Diškus and Stonis 2015).

In this current study, the expertise and specific interest in the documentation of leaf-mining Tischerioidea and Nepticuloidea of AD, JRS, AR, and MAS’s interest in large-scale Microlepidoptera taxonomy of the Americas and global faunas, and LK’s botanical expertise, particularly of Asteraceae taxonomy were combined.

The main goal of this publication is to describe seven new species of trumpet moths, possessing unusual genitalic characters, in order to have their names and biological data available for further analysis. We also identified previously unidentified Neotropical material from the collection holdings of the National Museum of Natural History (USNM). Further, we discovered that *Paratischeria neotropicana* (Diškus and Stonis 2015), which was already known to possess the broadest distribution among the Neotropical Tischeriidae, has an even broader distribution in Central and South America. We provide new host-plant data for Neotropical Tischeriidae, as well as a record of Sapindaceae, a new host-plant family for Tischeriidae worldwide, and, for the first time, a full list of host plants of the Neotropical Tischeriidae. We hypothesize that host-plant ranges predict a much broader distribution for host-specific leaf miners treated here through their host-plant distribution. Finally, we provide a short review on the history of Tischeriidae species descriptions in the Neotropics. We hope that this publication will stimulate further studies in Neotropical Tischeriidae and will contribute to a more detailed account of the diversity of the Neotropical leaf-mining insects.

## Materials and methods

The description of *Paratischeria braziliensis* sp. nov. is based on material deposited in the collection of the National Museum of Natural History (NMNH), formerly the U.S. National Museum of Natural History, Washington D.C., U.S.A. (USNM). The type series of six new species will be deposited at the Zoological Institute of the Russian Academy of Sciences, St. Petersburg, Russia (ZIN). New distribution data of *Paratischeria neotropicana* are based on the material from the collections of USNM, ZIN, the Zoological Museum, Natural History Museum of Denmark, University of Copenhagen, Copenhagen (ZMUC), and the Natural History Museum, London, U.K. (NHMUK).

Detailed techniques of rearing adults from mining larvae are provided by Diškus and Stonis (2012) and Stonis et al. (2018a). Protocols for species identification and description were outlined in Puplesis and Diškus (2003) and Stonis et al. (2014, 2018a). Permanent mounts on microscope slides were photographed and studied using a Leica DM2500 microscope and Leica DFC420 digital camera. Adults were photographed using a Leica S6D stereoscopic microscope with attached Leica DFC290 digital camera.

The descriptive terminology of morphological structures follows Puplesis and Diškus (2003), except for the term “aedeagus”, which is referred to here as “phallus”, and the term “cilia”, which is referred to here as “fringe”.

## Taxonomic accounts

### Astrotischeria jociui

Taxon classificationAnimaliaLepidopteraTischeriidae

Diškus & Stonis sp. nov.

D85686D0-B407-5D80-8227-694ECDBBA0D8

http://zoobank.org/FB1D5882-9D68-4613-9E67-6CFDABED85F8

Figs 15–18 , 38 , 39 , 50–59 , 60–67

#### Holotype.

male, pinned, with genitalia slide AD999. Labels: Peru, Urubamba Province, near Machu Picchu, 13°9'48"S, 72°32'10"W, elevation 2160 m, mining larva on *Wissadula* sp. (Malvaceae), 19 Oct 2008, field card no. 4945, A. Diškus (ZIN).

#### Diagnosis.

Externally, this new species can be confused with some other speckled *Astrotischeria* species, including the species described below. In the male genitalia, the unique shape of the bifid dorsal processes of valva (Figs 51, 56, 57) and the unusually complex, angular apex of phallus with ventral spines (Figs 52–55, 59) distinguishes *A. jociui* sp. nov. from all known congeneric species. In the female genitalia, the combination of wide processes of the prela (Figs 61, 63) and proximally very long and slender corpus bursae differentiate the new species from other *Astrotischeria* taxa. This species is also distinctive because no other species in this genus is known to feed on *Wissadula* Medik. (Malvaceae), except for the South American *Astrotischeria ochrimaculosa* Diškus, Stonis & Vargas, which possesses very different male genitalia (see Stonis et al. 2019b).

#### Description.

**Male** (Fig. 38). Forewing length 3.5–3.8 mm; wingspan 7.7–8.1 mm (n = 2). Head: frons and pecten ochre; frontal tuft and collar comprised of ochre and grey, ochre-tipped scales; antenna longer than one half the length of forewing; flagellum ochre, annulated with grey scales in proximal quarter, but grey distally. Tegula and thorax covered with ochre and grey, ochre-tipped scales. Forewing ochre to pale ochre, apically speckled with grey, ochre-tipped scales; fringe grey; forewing underside dark brown-grey, without spots or androconia. Hindwing and fringe grey on upper side and underside, without androconia. Legs dark brownish grey, with some ochre scales, especially numerous on underside and tarsi. Abdomen grey with some green and purple iridescence on upper side, brownish grey, with some pale ochre scales on underside; genital plates pale grey; anal tufts long, dark grey.

***Male genitalia*** (Figs 50–60) with capsule 870–950 µm long, 500–510 µm wide. Uncus (Figs 50, 56) comprised of two long, slender lobes. Socii small, paired, membranous. Valva (Figs 50, 51, 56–58) ca. 610–620 µm long (excluding the basal process); dorsal lobe (Figs 51, 56) greatly developed, bifid, curved dorsally; ventral lobe of valva with a unique spine-like process (Figs 56, 57, 60). Anellus mostly membranous, thickened only laterally (Fig. 51). Vinculum rounded distally (Fig. 50). Phallus (Fig. 59) ca. 505–520 µm long, apically very complex, angular (Fig. 55), with a pair of spine-like processes (Figs 52–54).

**Female** (Fig. 39). Forewing length 2.8–3.1 mm; wingspan 6.2–6.8 mm (n = 2). Head similar to male, but frons and palpus pale ochre. Thorax similar to male, but thorax and forewing tend to be slightly darker and dark scales less contrast to main color of forewing. Abdomen similar to male, but without anal tufts, and with a protruding slender ovipositor.

***Female genitalia*** (Figs 61–67) ca. 2550 µm long. Ovipositor lobes large (Figs 63–65), clothed with peg-like setae. Posterior apophyses slightly shorter than anterior ones (Figs 61, 63); prela comprised of three pairs of unique projections (Figs 61, 63). Corpus bursae with very slender and long (1020 µm) proximal part (Fig. 62) and oval main body (Fig. 67); pectination indistinctive. Ductus spermathaecae with many large coils (Fig. 66).

#### Bionomics.

(Figs 15–18). Host plant is *Wissadula excelsior* (Cav.) C. Presl., Malvaceae (Fig. 15). Larvae mine leaves in October. The blotch-like mine (Figs 17, 18) is irregular, usually white, fully transparent, without frass. Adults occur in late October – November.

#### Distribution.

This species is known from a single locality in Peru, Urubamba Province, near Machu Picchu, at the elevation 2000–2200 m (Fig. 16), but the host plants have a much wider distribution (see Discussion).

#### Etymology.

The species is named in honor of Mr. Modestas Jocius (Vilnius, Lithuania), recognizing his understanding, continued support, and enthusiasm for biodiversity inventories in tropical countries.

#### Other material examined.

4 ♂, 4 ♀, paratypes: Peru, Urubamba Province, near Machu Picchu, 13°9'48"S, 72°32'10"W, elevation 2160 m, mining larvae on *Wissadula* sp. (Malvaceae), 19 Oct 2008, field card no. 4945, A. Diškus, genitalia slide nos AD922♂ (from adult in pupal skin, no moths preserved), AD976♂ (from adult in pupal skin, no moths preserved), AD997♀ (from adult in pupal skin, no moths preserved), AD977♂ (from adult in pupal skin, no moths preserved), AD978♀ (ZIN).

### Astrotischeria atlantica

Taxon classificationAnimaliaLepidopteraTischeriidae

Diškus & Stonis sp. nov.

84D2CF54-663C-545B-A6CE-0DA87316EE77

http://zoobank.org/24A33F1D-005A-4BB1-AF77-56D5543BD528

Figs 1–9 , 40 , 41 , 68–76 , 77–80

#### Holotype.

male, pinned, with genitalia slide no. AD969. Labels: Uruguay, Rocha Department, La Paloma, 34°39'41"S, 54°13'4"W, elevation 5 m, mining larva on *Baccharis spicata* (Lam.) Baill., Asteraceae, 26 Feb 2019, field card no. 5303, A. Diškus (ZIN).

**Figures 1–9.**
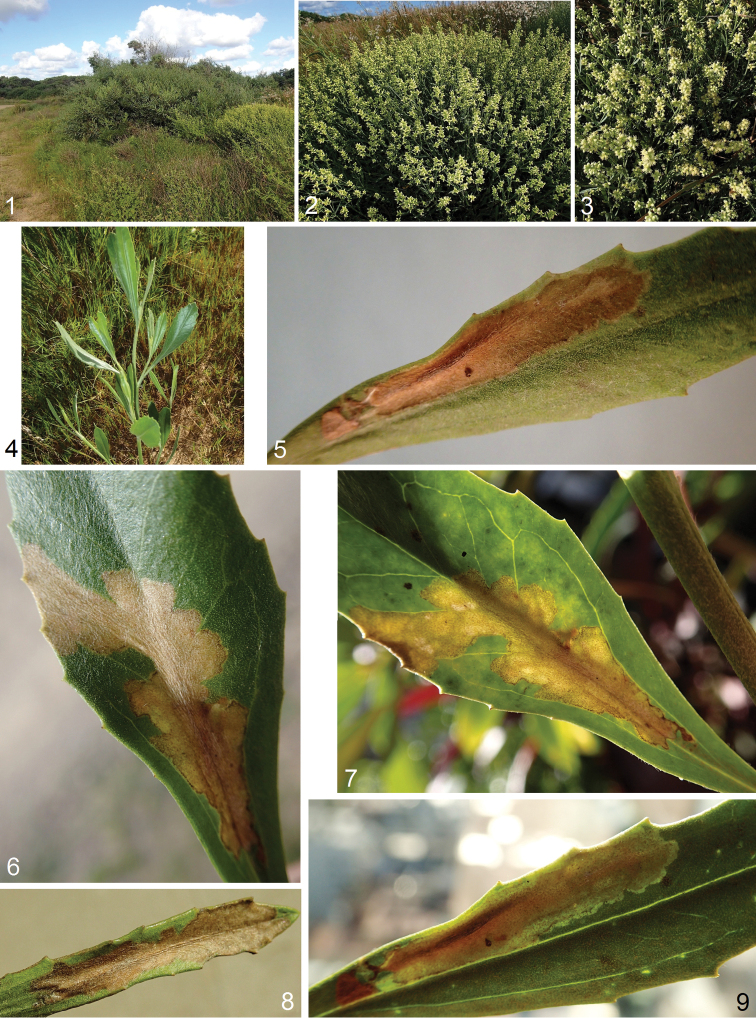
Bionomics of *Astrotischeria atlantica* sp. nov. **1** habitat, elevation 5 m, La Paloma, Rocha Department, Uruguay **2–4** host plant *Baccharis spicata* (Lam.) Baill., Asteraceae**5–9** leaf mines.

#### Diagnosis.

Externally, this new species can be confused with some other speckled *Astrotischeria* species, including the species described in this paper. *Astrotischeria atlantica* sp. nov. can be distinguished from similar *A. jociui* sp. nov. (see described above) by the significantly paler color of forewing: in *A. atlantica* forewing is cream to pale yellowish ochre, in *A. jociui* is ochre. In the male genitalia, the shape of dorsal processes of the valva with unique folds (Fig. 75) and the presence of additional lobes on the uncus (Figs 68, 69) distinguish *A. atlantica* sp. nov. from all known congeneric species. In the female genitalia, the presence of a highly modified ovipositor (Fig. 78) differentiates this new species from other *Astrotischeria* taxa. This species is also distinctive because no other species in this genus is known to feed on *Baccharis spicata* (Lam.) Baill. (Asteraceae).

#### Description.

**Male** (Fig. 40). Forewing length 3.6–4.2 mm; wingspan 7.7–9.3 mm (n = 2). Head: frons and pecten golden cream; frontal tuft glossy cream distally, ochre-grey proximally; collar ochre-grey; antenna slightly longer than one half the length of forewing; flagellum yellowish cream proximally, pale yellowish grey distally. Tegula yellowish grey, distally cream; thorax yellowish cream. Forewing cream to pale yellowish ochre, irregularly speckled with grey and pale grey scales, apically also with some black scales; fringe pale grey, with fringe line comprised of black scales; forewing underside pale ochre-grey to brownish cream, without spots or androconia. Hindwing glossy greyish cream to cream on upper side, pale grey on underside, without androconia, but sometimes with a dark line of grey scales along one third of the fold; fringe cream. Foreleg pale grey or blackish grey on upper side, midleg and hindleg ochre cream to cream, with some pale grey scales on upper side and spurs. Abdomen yellow cream, distally pale grey on upper side, pale ochre with some grey scales on underside; genital plates large, covered with long, yellow cream scales; anal tufts long, merged into one, cream.

***Male genitalia*** (Figs 68–76) with capsule 1120 µm long, 730 µm wide. Uncus (Figs 68–71) comprised of two short, triangular lobes (Fig. 71) and two long, slender lobes (Fig. 70); the latter possess a unique lobe-like process (Figs 68, 69). Socii small, paired, membranous. Valva (Figs 72, 75) ca. 730 µm long; dorsal lobe (Figs 72, 75) greatly developed, with folds distally (Fig. 75); ventral lobe of valva slender. Anellus mostly membranous, thickened only laterally (Fig. 75). Vinculum rounded distally (Fig. 72). Phallus ca. 970 µm long, apically bifid, with hook-like apices (Fig. 73).

**Female** (Fig. 41). Forewing length 3.6–4.3 mm; wingspan 7.7–9.4 mm (n = 2). Similar to male, but thorax and forewing tend to be paler, i.e., less speckled with grey scales. Anal tuft long, ochre cream; ovipositor slightly protruding. Otherwise, identical with male.

***Female genitalia*** (Figs 77–80) ca. 3410 µm long. Ovipositor lobes modified into a unique (among Tischeriidae) plate-like ovipositor without peg-like setae (Fig. 78); second pair of ovipositor lobes large, with numerous long setae. Posterior apophyses shorter than anterior ones (Fig. 79); prela comprised of three pairs of unique projections (Fig. 79). Corpus bursae with very slender and long proximal part and small main body without pectination (Fig. 77). Ductus spermathaecae with three large coils (Fig. 77).

#### Bionomics.

(Figs 1–9). Host plant is *Baccharis spicata* (Lam.) Baill., Asteraceae (Figs 1–4). Larvae mine leaves in February. The blotch-like mine (Figs 5–9) is irregular, but elongated, pale brown or pale green, without frass. Adults occur in March.

#### Distribution.

This species is known from a single locality on the Atlantic coast in Uruguay, Rocha Department, La Paloma (Fig. 1), at sea level, but the host plant has a much wider distribution (see Discussion).

#### Etymology.

The species is named after the Atlantic Ocean, in reference to its occurrence on the Atlantic coast of Uruguay.

#### Other material examined.

2 ♂, 3 ♀, paratypes: Uruguay, Rocha Department, La Paloma, 34°39'41"S, 54°13'4"W, elevation 5 m, mining larvae on *Baccharis spicata* (Lam.) Baill., Asteraceae, 26 Feb 2019, field card no. 5303, A. Diškus, genitalia slide nos AD970♂ (from adult in pupal skin, no moths preserved), AD968♀ (ZIN).

### Astrotischeria cornuata

Taxon classificationAnimaliaLepidopteraTischeriidae

Diškus & Stonis sp. nov.

99801396-A5D0-5BC0-AB58-0F450B580F76

http://zoobank.org/3ECCBA77-7B14-44F4-9A81-0331ADABAB6C

Figs 10–14 , 42 , 43 , 81–88 , 89–91

#### Holotype.

male, pinned, with genitalia slide no. AD522. Labels: Honduras, Copán Department, Copán, 14°50'13"N, 89°8'37"W, elevation 620 m, from feeding larva (Asteraceae host plant unidentified), 15 Feb 2012, field card no. 5090, A. Diškus (ZIN).

**Figures 10–14.**
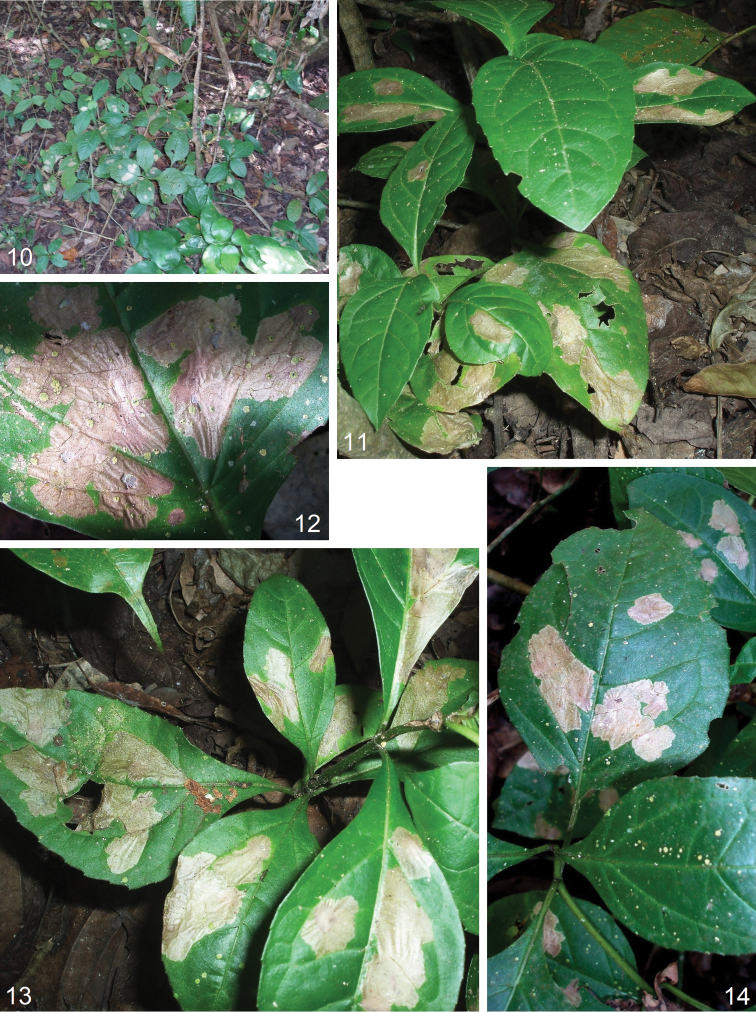
Bionomics of *Astrotischeria cornuata* sp. nov. **10** habitat, elevation 620 m, Copán, Copán Department, Honduras **11–14** leaf mines on unidentified Asteraceae host plant.

#### Diagnosis.

Externally, this new species can be confused with some other dark speckled Tischeriidae species, including *Paratischeria mesoamericana* sp. nov. (described below). In the male genitalia, the presence of pseudotranstilla (Figs 83, 87) and a unique, four-furcated phallus (Figs 85, 86) distinguish *A. cornuata* sp. nov. from all known congeneric species. In the female genitalia, the combination of large ovipositor lobes, densely covered with peg-like setae (Fig. 90) and a very small corpus bursae (Fig. 89) distinguish the new species from other *Astrotischeria* taxa.

#### Description.

**Male** (Fig. 42). Forewing length 2.8–3.2 mm; wingspan 6.0–7.2 mm (n = 3). Head: frons grey cream to yellow-ochre; pecten golden pale grey to ochre cream; frontal tuft glossy, metallic grey, distally yellow-ochre; collar yellow-ochre; antenna slightly to distinctly longer than one half the length of forewing; flagellum glossy grey. Tegula grey; thorax grey-ochre medially, grey laterally and distally. Forewing densely irrorated with dark grey scales (in apical half of the forewing majority of these scales are ochre-tipped) and with irregular, oblique patches of bright yellow-ochre scales; fringe dark grey, apically ochre; fringe line distinctive, comprised of dark grey scales; forewing underside blackish grey, without spots or androconia. Hindwing dark grey or black-grey depending on angle of view, without androconia; fringe dark grey with some ochreous-purple tint. Legs dark grey or black-grey, irregularly annulated with ochre cream scales on upper side. Abdomen glossy dark grey-brown on upper side and underside, sometimes with some purple iridescence; genital plates ochreous cream; anal tufts cream to grey cream: two dorsal tufts large, almost merged in one, lateral tufts shorter.

***Male genitalia*** (Figs 81–88) with capsule 880–920 µm long, 410–445 µm wide. Uncus (Figs 81–83) comprised of two short, widely rounded lobes (Fig. 81) and two long, slender lobes (Figs 82, 83). Socii small, paired, membranous. Valva (Figs 83, 84) ca. 780–790 µm long; dorsal lobe (Fig. 83) greatly developed, slender, curved inwardly (Fig. 88); ventral lobe of valva very slender and straight (Figs 83, 84). Valvae connected with a unique transverse band which we call here a pseudotranstilla (Figs 83, 87). Anellus mostly membranous, indistinctive (Fig. 83). Vinculum rounded distally (Fig. 83). Phallus (Fig. 86) ca. 475–485 µm long, apically split in two short, weakly chitinized, median lobes and two pointed, lateral lobes, the latter each with an apical spine (Fig. 85).

**Female** (Fig. 43). Forewing length 3.0–3.5 mm; wingspan 6.6–7.6 mm (n = 4). Similar to male, but with a yellow-ochre postmedian area of forewing, usually larger and often resembling a fascia. Abdomen dark grey on upper side, yellow-ochre with some grey scales on underside. Ovipositor not protruding.

***Female genitalia*** (Figs 89–91) ca. 3150 µm long. Ovipositor lobes unusually large, rounded, densely covered with peg-like setae (Fig. 90); second pair of ovipositor lobes very small and slender, with numerous long setae. Anterior and posterior apophyses equal in length (Fig. 90); prela comprised of three pairs of unique, rod-like projections (Fig. 91). Corpus bursae very long, with a slender proximal part and very small main body without distinctive pectination (Fig. 89). Ductus spermathaecae sinuous, without distinctive coils (Fig. 89).

#### Bionomics.

(Figs 10–14). Host plant is an Asteraceae, genus and species unidentified (Figs 10, 11). Larvae mine leaves in February. The mine is blotch-like (Figs 11–14), irregularly shaped, whitish grey, not transparent, without frass. Adults occur in March.

#### Distribution.

This species is known from a single locality in Honduras, Copán Department, Copán, at the elevation of 620 m.

#### Etymology.

The species name is derived from Latin *cornuatus* (horned), in reference to the large, horn-like lobes of the uncus and valva in the male genitalia.

#### Other material examined.

2 ♂, 4 ♀, paratypes: Honduras, Copán Department, Copán, 14°50'13"N, 89°8'37"W, elevation 620 m, from feeding larvae (Asteraceae host plant unidentified), 15 Feb 2012, field card no. 5090, A. Diškus, genitalia slide nos AD975♂, AD981♀ (ZIN).

**Figures 15–21.**
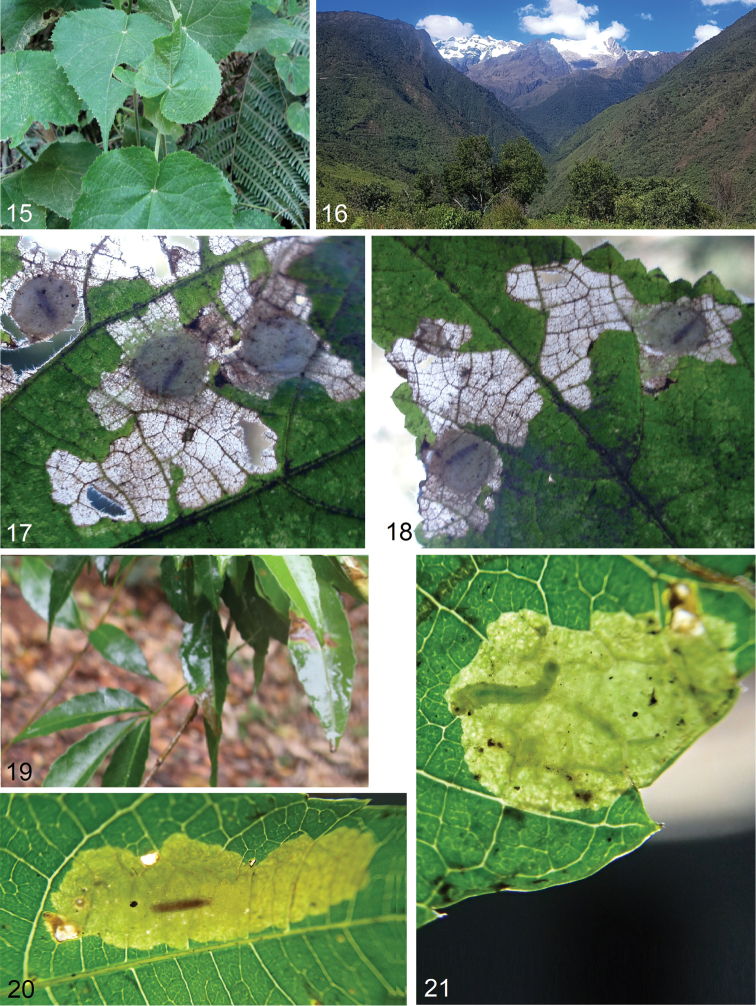
Bionomics of new species. **15***Astrotischeria jociui* sp. nov., host plant *Wissadula excelsior* (Cav.) C. Presl., Malvaceae**16** same, habitat, elevation 2160 m, near Machu Picchu, Urubamba Province, Peru **17, 18** same, leaf mines on *Wissadula excelsior***19***Paratischeria suprafasciata* sp. nov., host plant *Allophylus edulis* (A. St.-Hil., A. Juss. & Cambess.) Hieron. ex Niederl., Sapindaceae**20** leaf mine with a pupa **21** leaf mine with a feeding larva.

### Paratischeria guarani

Taxon classificationAnimaliaLepidopteraTischeriidae

Diškus & Stonis sp. nov.

278131A7-7D26-5262-811A-F6BCFA49453A

http://zoobank.org/D3990BF8-93B3-4843-BEC8-A38F06888465

Figs 22–31 , 44 , 45 , 92–96

#### Holotype.

male, pinned, with genitalia slide no. AD988. Labels: Paraguay, Departamento de Itapúa, Hohenau, 27°5'6"S, 55°40'22"W, elevation 115 m, mining larva on *Elephantopus mollis* Kunth, Asteraceae, 14 Feb 2019, field card no. 5293, A. Diškus (ZIN).

**Figures 22–31.**
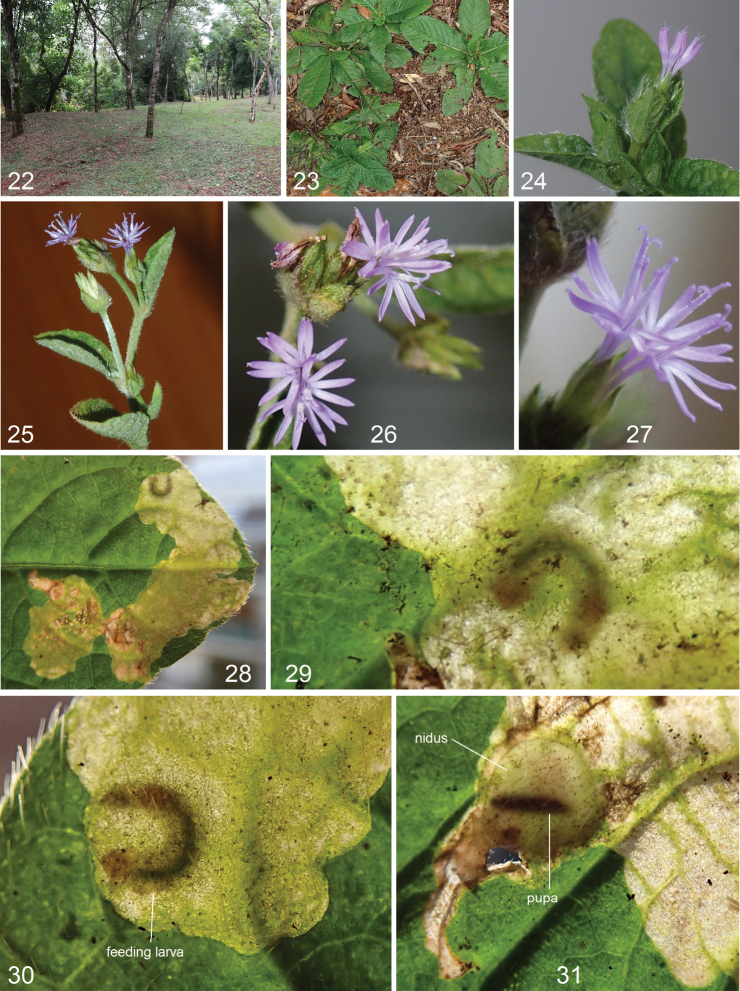
Bionomics of *Paratischeria guarani* sp. nov. **22** habitat, elevation 115 m, Hohenau, Departamento de Itapúa, Paraguay **23–27** host plant *Elephantopus mollis* Kunth, Asteraceae**28–30** leaf mines with a feeding larva **31** leaf mine with a pupa.

#### Diagnosis.

Externally, this new species can be confused with some other brightly colored species, including *A. cornuata* sp. nov. (described above) or Central American *A. guatemalica* Diškus & Stonis, and Ecuadorian *A. bachariphaga* Diškus & Stonis (see Stonis et al. 2019). However, these externally similar *Astrotischeria* species possess a well-developed dorsal lobe of valva, but all *Paratischeria* species have no dorsal lobe. In the male genitalia, the combination of very long and slender uncus and a laterally strongly thickened anellus distinguish *Paratischeria guarani* sp. nov. from all known congeneric species. The characters of the female genitalia are not informative, and, therefore, are of very limited use for species differentiation. This species is also distinctive because no other tischeriid species is known to feed on *Elephantopus mollis* Kunth, Asteraceae.

#### Description.

**Male** (Fig. 44). Forewing length 2.8–3.1 mm; wingspan 6.1–6.8 mm (n = 4). Head: frons ochre-grey, pecten pale ochre; frontal tuft glossy grey proximally, pale ochre distally; collar ochre-grey; antenna slightly longer than one half length of forewing; flagellum greyish cream, irregularly annulated with dark grey scales. Tegula and thorax grey-ochre. Forewing variable, pale ochre irregularly speckled with dark grey, apically with black scales; fringe dark grey, with fringe indistinctive or absent; forewing underside brown-black, without spots or androconia. Hindwing grey on upper side and underside, without androconia; fringe grey. Legs grey on upper side, ochre cream on underside. Abdomen black on upper side, glossy ochre with some dark brown scales on underside; genital plates pale ochre to pale grey; anal tufts grey-ochre.

***Male genitalia*** (Figs 92–94) with capsule 760 µm long, 315 µm wide. Uncus (Fig. 93) with two very long and slender lateral lobes. Socii small, paired, membranous. Valva (Fig. 92) ca. 615 µm long. Anellus strongly thickened laterally, constricted at the middle (Fig. 92). Vinculum small, rounded distally (Fig. 92). Phallus ca. 670 µm long, apically bifid (Fig. 94).

**Female** (Fig. 45). Forewing length 3.0–3.3 mm; wingspan 6.5–7.1 mm (n = 2). Similar to male, but sometimes darker, with bright ochre thorax and forewing more intensively speckled with black scales. Abdomen black, ochre only distally; ovipositor very short, but protruding. Otherwise, identical with male.

***Female genitalia*** (Figs 95–97) 680 µm long. Ovipositor lobes oval-shaped, covered with peg-like setae; second pair of ovipositor lobes small, with long setae (Fig. 96). Anterior and posterior apophyses equal in length (Fig. 96); prela comprised of three pairs of rod-like projections (Fig. 96). Corpus bursae very slender, with long proximal part and small main body without pectination (Fig. 95). Ductus spermathecae with 7–8 large coils (Fig. 97).

#### Bionomics

(Figs 22–31). Host plant is *Elephantopus mollis* Kunth, Asteraceae (Figs 23–27). Larvae mine leaves in February. Larva greenish white, with dark green intestine and brown head. The blotch-like mine (Figs 28–31) is irregular, but often elongated, pale brown or pale green, without frass. Pupation in a round nidus. Adults occur in March.

#### Distribution.

This species is known from a single locality in Paraguay, Departamento de Itapúa, Hohenau (Fig. 22), at the elevation of 115 m, but the host plant has a much wider distribution (see Discussion).

#### Etymology.

This species is named after the Guaraní, indigenous people of South America, living in present-day Paraguay between the Uruguay River and lower Paraguay River.

#### Other material examined.

5 ♂, 2 ♀, paratypes: Paraquay, Departamento de Itapúa, Hohenau, 27°5'6"S, 55°40'22"W, elevation 115 m, mining larvae on *Elephantopus mollis* Kunth, Asteraceae, 14 Feb 2019, field card no. 5293, A. Diškus, genitalia slide nos AD986♂ (from adult in pupal skin, no pinned moth preserved), AD998♂, AD987♀ (ZIN).

### Paratischeria mesoamericana

Taxon classificationAnimaliaLepidopteraTischeriidae

Diškus & Stonis sp. nov.

896F4367-A0D4-5005-9B5B-DDAA29926041

http://zoobank.org/A1306212-7A99-4237-84BB-D2A138A2A013

Figs 32–37 , 46 , 48 , 98–104 , 105–110

#### Holotype.

male, pinned, with genitalia slide no. AD1005. Labels: Guatemala, Antigua Guatemala, San Juan del Obispo, 14°31'7"N, 90°43'50"W, elevation 1680 m, feeding larva on *Montanoa hibiscifolia* Benth., Asteraceae, 25 Feb 2012, field card no. 5109, A. Diškus (ZIN).

**Figures 32–37.**
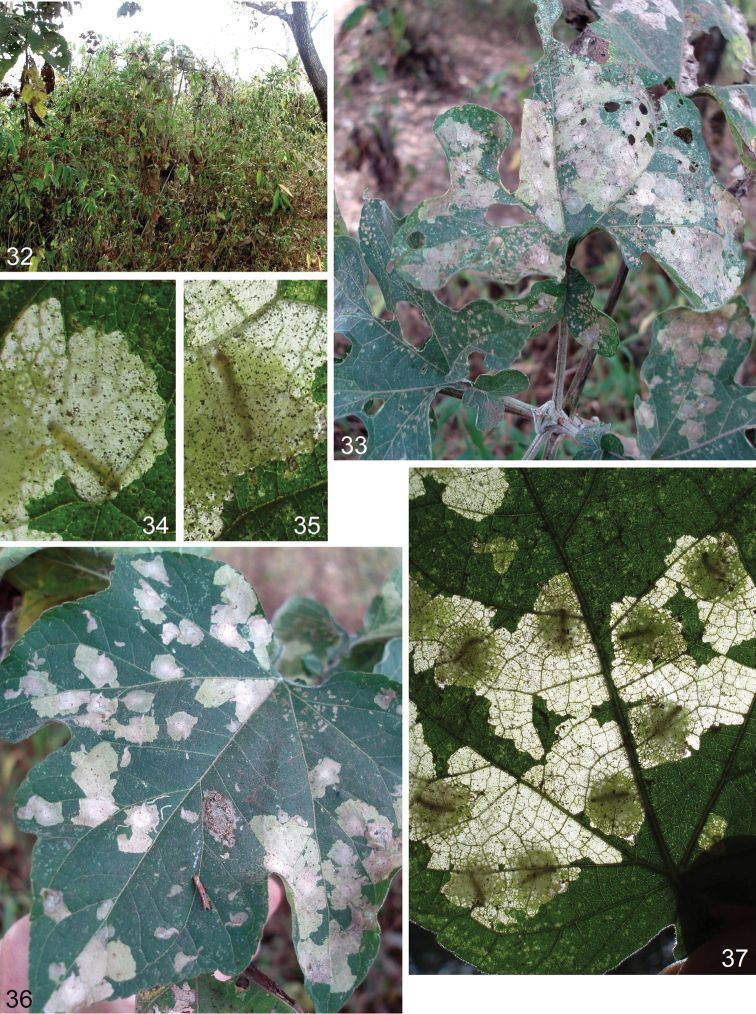
Bionomics of *Paratischeria mesoamericana* sp. nov. **32** habitat and host plant *Montanoa hibiscifolia* Benth., Asteraceae, elevation 1680 m, San Juan del Obispo, Antigua Guatemala, Guatemala **33–37** leaf mines with feeding larvae.

#### Diagnosis.

Externally, *P. mesoamericana* sp. nov. can be confused with some brightly speckled *Astrotischeria* Puplesis & Diškus species, including *A. cornuata* sp. nov. (described above) or the Central American *A. guatemalica* Diškus & Stonis, South American *A. bachariphaga* Diškus & Stonis, and *A. truncata* Diškus & Stonis (in Stonis et al. 2019). However, all these externally similar species belong to another genus, *Astrotischeria*, and possess principally different male genitalia with dorsal lobe(s) on the valva. In the male genitalia, the combination of a unique, distally pointed, four-lobed phallus (Fig. 99), medially constricted anellus (Fig. 98), and the presence of bifid cheatae on the valva (Fig. 101) distinguish *P. mesoamericana* sp. nov. from all known congeneric species. This species is also distinctive because no other species in this genus is known to feed on *Montanoa hibiscifolia* Benth., Asteraceae.

#### Description.

**Male** (Fig. 46). Forewing length 2.6–3.8 mm; wingspan 5.7–8.6 mm (n = 10). Head: frons and pecten ochreous cream; frontal tuft ochre cream, distally whitish cream; collar ochre cream; antenna longer than one half the length of forewing; flagellum glossy cream, usually annulated with dark brown or pale brown scales. Tegula ochre cream, densely irrorated with grey-brown scales; thorax ochre cream. Forewing yellow-ochre with irregular patches of dark scales: most of these scales are cream but black-tipped, others are plain brown; fringe grey, apically ochre; fringe line present, sometimes ill-defined, comprised of brown and black-tipped cream scales; forewing underside ochre-brown, apically sometimes brownish cream, without spots or androconia. Hindwing glossy, pale grey on upper side and underside, at base cream; fringe pale grey. Legs covered with grey-brown scales on upper side, ochreous cream on underside. Abdomen glossy, pale grey to grey-brown depending on angle of view, with some purple iridescence on upper side, ochre cream, densely speckled with grey-brown or ochre-brown scales on underside; genital plates ochre cream; anal tufts long, dorsally paired, cream.

***Male genitalia*** (Figs 98–104) with capsule 890 µm long, 450 µm wide. Uncus comprised of two short, rounded lobes (Fig. 100) and two long, slender lobes (Fig. 104). Socii small, paired, membranous. Valva (Figs 98, 102) ca. 660 µm long, with bifid chaetae. Anellus thickened laterally and constricted medially (Figs 98, 103). Vinculum small, rounded distally (Fig. 102). Phallus (Fig. 99) ca. 675 µm long, apically split into four pointed lobes.

**Female** (Fig. 43). Forewing length 3.2–4.0 mm; wingspan 7.0–8.7 mm (n = 8). Scaling similar to male, but sometimes can be darker; frontal tuft ochre cream to ochre-brown. Thorax ochre cream to ochre-brown. Forewing sometimes darker than in males. Abdomen ochre-brown to brown, with some purple iridescence on upper side, ochre cream densely covered with brown or dark brown scales on underside. Ovipositor protruding.

***Female genitalia*** (Figs 105–110) 2520–2680 µm long. Ovipositor lobes unusually small, rounded, with peg-like setae (Fig. 109); second pair of ovipositor lobes only slightly smaller. Anterior apophyses slightly shorter than posterior apophyses (Fig. 107); prela with three pairs of long, rod-like projections (Figs 109, 110). Corpus bursae with a very slender but very long proximal part, and a small, oval, main body (Fig. 107); pectination indistinctive. Ductus spermathecae with many large coils (Fig. 108).

#### Bionomics.

(Figs 32–37). Host plant is *Montanoa hibiscifolia* Benth., Asteraceae (Figs 32, 33). Larvae mine leaves in February. Larva is greenish yellow with a brownish green intestine and brown head. The mine is blotch-like (Figs 33–37), usually slightly angular, fully transparent, without frass. Adults occur in March.

#### Distribution.

This species is known from a single locality in Guatemala: Antigua Guatemala, San Juan del Obispo, at the elevation 1680 m, but the host plant has a much wider distribution (see Discussion).

#### Etymology.

The species named after Mesoamerica, a historical region of North America.

#### Other material examined.

14 ♂, 27 ♀, paratypes: Guatemala, Antigua Guatemala, San Juan del Obispo, 14°31'7"N, 90°43'50"W, elevation 1680 m, feeding larvae on *Montanoa hibiscifolia* Benth., Asteraceae, 25 Feb 2012, field card no. 5109, A. Diškus, genitalia slide nos AD871♂, AD887♀, AD1006♀ (ZIN).

**Figures 38–43.**
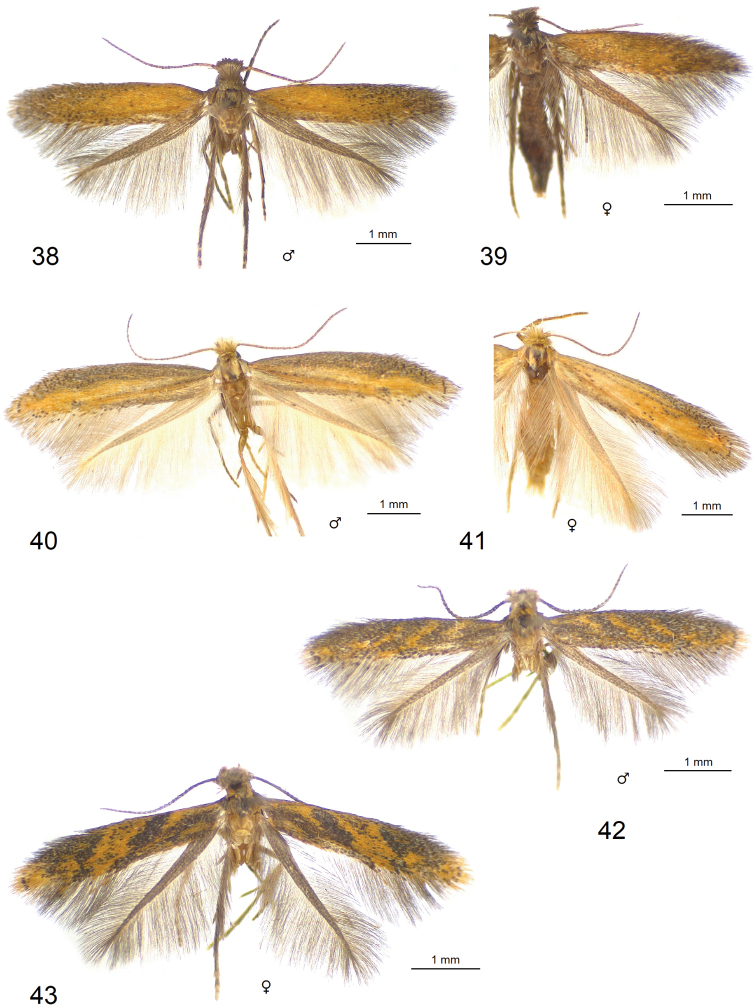
Adults of *Astrotischeria* spp. **38***A. jociui* sp. nov., male, holotype **39** same, female, paratype **40***A. atlantica* sp. nov., male, holotype **41** same, female, paratype **42***A. cornuata* sp. nov., male, holotype **43** same, female, paratype (ZIN).

### Paratischeria suprafasciata

Taxon classificationAnimaliaLepidopteraTischeriidae

Diškus & Stonis sp. nov.

718552E3-EAF8-54CB-AEE2-2132F5D778F6

http://zoobank.org/DA9C53D0-66CB-4FDB-B116-711B7AECF3B3

Figs 19–21 , 47 , 111–115

#### Holotype.

female, pinned, with genitalia slide no. AD967. Labels: Argentina, Misiones Province, Puerto Iguazú, 25°41'8"S, 54°26'47"W, elevation 160 m, mining larva on *Allophylus edulis* (A. St.-Hil., A. Juss. & Cambess.) Hieron. ex Niederl., Sapindaceae, 10 Feb 2019, field card no. 5291, A. Diškus (ZIN).

**Figures 44–49.**
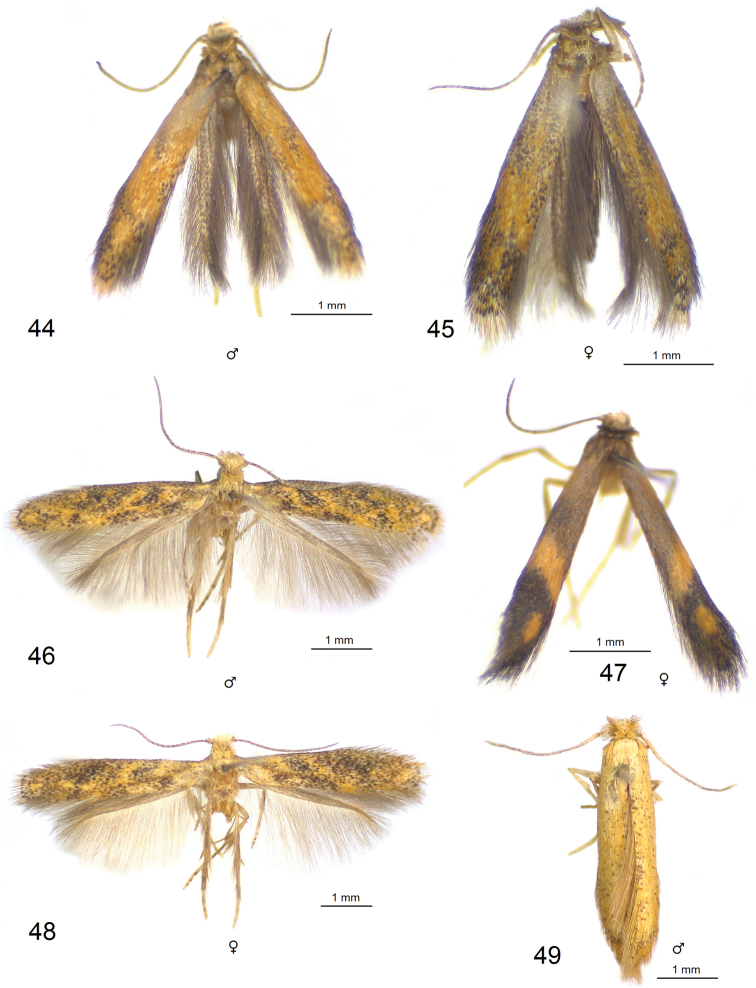
Adults of *Paratischeria* spp. **44***P. guarani* sp. nov., male, holotype (ZIN) **45** same, female, paratype (ZIN) **46***P. mesoamericana* sp. nov., male, holotype (ZIN) **47***P. suprafasciata* sp. nov., female, holotype (ZIN) **48***P. mesoamericana* sp. nov., female, paratype (ZIN) **49***P. braziliensis* sp. nov., male, holotype (USNM).

#### Diagnosis.

Externally, this new species can be differentiated from all congeneric species by the distinct forewing pattern with an ochre, oblique, postmedian fascia and ochre subapical spot (Fig. 47). Male unknown. In the female genitalia, the new species is characterized by the unique, oval-shaped widening of slender part of corpus bursae proximally (Fig. 111). This species is also distinctive because no other species in Tischeriidae is known to feed on *Allophylus edulis* (A. St.-Hil., A. Juss. & Cambess.) Hieron. ex Niederl. (Sapindaceae).

#### Description.

**Male.** Unknown.

**Female** (Fig. 47). Forewing length 3.2 mm; wingspan 6.9 mm (n = 1). Head: frons and pecten ochre cream to pale ochre; golden cream; collar glossy ochre-grey; antenna slightly longer than one half the length of forewing; flagellum dark grey on upper side, pale grey on underside. Tegula and thorax glossy ochre-grey. Forewing slender, glossy grey-ochre in basal half, with bright ochre, oblique postmedian fascia and bright ochre subapical spot widely surrounded by black and cream-tipped scales with purple iridescence; fringe black-grey, without fringe line. Hindwing and fringe grey on upper side and underside, without androconia. Legs grey to blackish grey on upper side, ochre cream on underside. Abdomen blackish grey on upper side, glossy pale ochre with some grey scales (especially prominent proximally) on underside; anal tufts absent; ovipositor slightly protruding.

***Female genitalia*** (Figs 111–115) 1310 µm long. Ovipositor lobes large, rounded, clothed with short, modified peg-like setae; area between ovipositor lobes slender, with tiny papillae and some short setae. Second pair of lobes, lateral and anterior to the ovipositor lobes, slightly smaller, triangular, with long slender setae. Posterior apophyses slightly shorter than anterior ones (Fig. 115); prela comprised of three pairs of rod-like projections (Fig. 115); inner pair of these rod-like projections very long (as long as anterior apophyses). Corpus bursae folded, oval-shaped proximally, bulbous distally (Fig. 111), without pectination or signum on wide, basal part, but with some tiny spines proximally (Fig. 113). Ductus spermathaecae very slender, with many coils (Fig. 112).

#### Bionomics.

(Figs 19–21). Host plant is *Allophylus edulis* (A. St.-Hil., A. Juss. & Cambess.) Hieron. ex Niederl., Sapindaceae (Fig. 19). Larvae mine leaves in February. Larva very pale green with a bright green intestine and very pale brown head. The mine is blotch-like (Figs 20, 21), fully transparent, without frass. Adults occur in March.

#### Distribution.

This species is known from a single locality in northern Argentina, Misiones Province, Puerto Iguazú, at the elevation ca. 160 m, but the host plant has a much wider distribution (see Discussion).

#### Etymology.

The species name is derived from Latin *fasciatus* (banded, with a fascia) with the prefix *supra*, in reference to the unusual (in Tischeriidae), forewing pattern with a distinctive postmedian facia.

**Figures 50–59.**
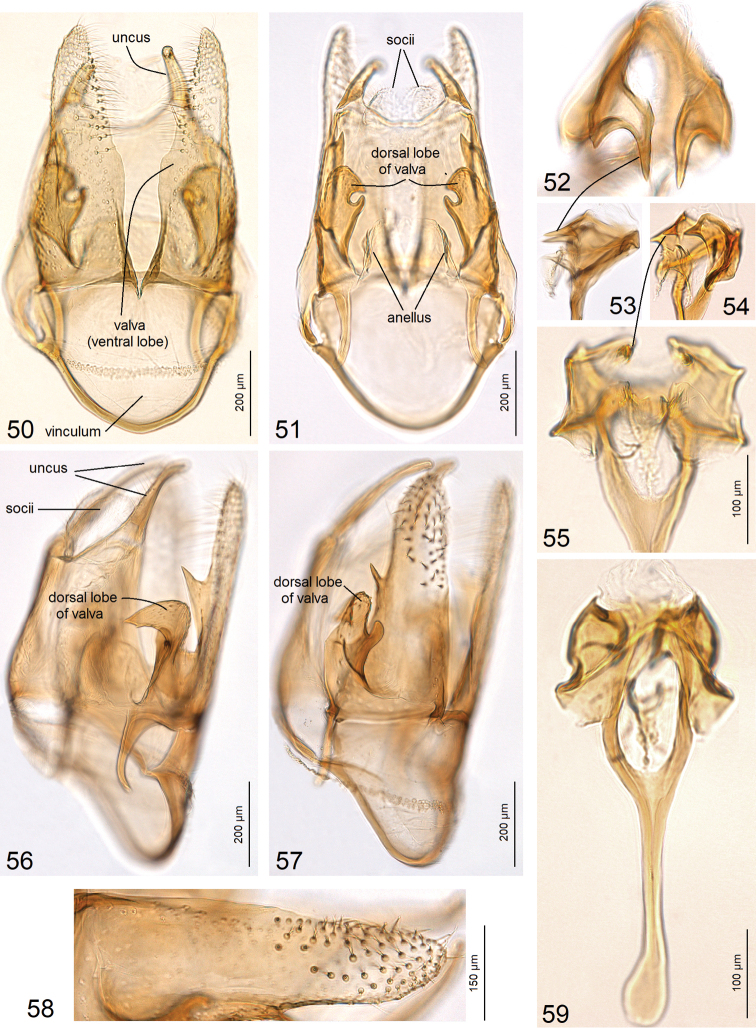
Male genitalia of *Astrotischeria jociui* sp. nov. **50, 51** capsule with phallus removed, holotype, genitalia slide no. AD999 **52, 53** apex of phallus, paratype, genitalia slide no. AD977 **54** same, genitalia slide no. AD976 **55** same, genitalia slide no. AD922 **56–58** lateral view of capsule, paratype, genitalia slide no. AD977 **59** phallus, general view, paratype, genitalia slide no. AD977 (ZIN).

### Paratischeria braziliensis

Taxon classificationAnimaliaLepidopteraTischeriidae

Diškus & Stonis sp. nov.

C7B1502C-71E8-5A46-A493-20902847B2C9

http://zoobank.org/E51DF3A6-2756-44EB-B824-14FF97EAC647

Figs 49 , 116–126

#### Holotype.

male, pinned, with genitalia slide no AD1004. Label: Brazil, Nova Teutônia, 27°11'S, 52°23'W, Oct 1944, Fritz Plaumann (USNM).

**Figures 60–67.**
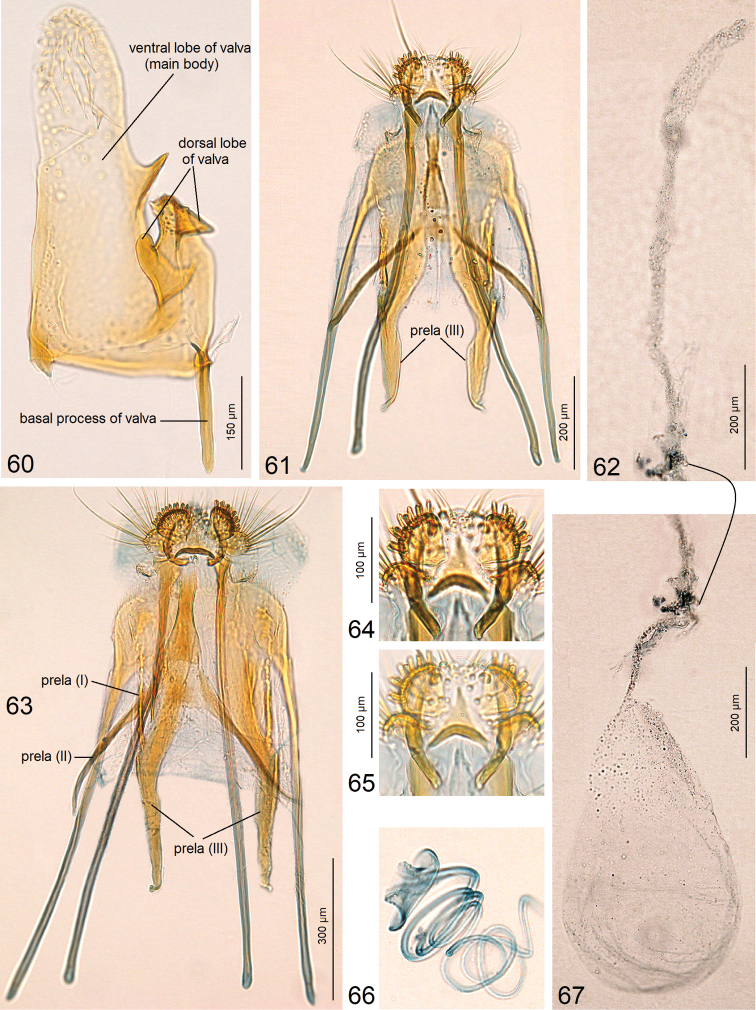
Genitalia of *Astrotischeria jociui* sp. nov. **60** male genitalia, valva, lateral view, paratype, genitalia slide no. AD976 **61, 63** female genitalia, paratype, genitalia slide no. AD978, ovipositor lobes and apophyses **62** same, slender part of corpus bursae **64, 65** same, details of ovipositor lobes **66** same, coils of ductus spermathecae **67** same, main body of corpus bursae (ZIN).

#### Diagnosis.

External characters are not informative for species identification: this new species can be confused with many other pale speckled *Paratischeria* Diškus & Stonis, *Coptotriche* Walsingham, and *Astrotischeria* Puplesis & Diškus species. In the male genitalia, the unique, unusually long, rod-like process of vinculum (Figs 120, 121), absence of transtilla, and the unique, spiny phallus (Figs 124–126) easily differentiate *P. braziliensis* sp. nov. from all known Tischeriidae species (also see Discussion).

#### Description.

**Male** (Fig. 38). Forewing length 3.9 mm; wingspan 8.4 mm (n = 1). Head: frons and pecten glossy whitish cream; frontal tuft ochre cream, but distally glossy whitish over the frons, laterally with some brown-tipped scales; collar ochre cream; antenna only longer than one half the length of forewing; flagellum glossy yellow cream. Tegula pale ochre-yellow; thorax ochre cream. Forewing pale ochre yellow, with irregularly scattered ochre-brown scales; fringe indistinct or absent; forewing underside pale ochre-brown. Hindwing and fringe yellow-ochre. Legs pale yellow ochre, with some ochre-brown scales on upper side. Abdomen ochre cream on upper side and underside; genital plates cream; anal tufts long, cream.

***Male genitalia*** (Figs 116–126) with capsule 550 µm long, 450 µm wide. Uncus (Fig. 118) comprised of two large, strongly thickened, lateral lobes. Socii membranous, unpaired, unusually large (Fig. 116). Valva (Figs 117, 119, 120) 500 µm long (excluding the basal process), wide (Fig. 119). Transtilla absent. Anellus indistinctive. Vinculum with unusually slender but very long (785 µm), rod-like process (Figs 120, 121). Phallus (Figs 122–126) 1185 µm long, apically with three lobes and some large spines laterally (Figs 124, 126).

**Female.** Unknown.

#### Bionomics.

Adults fly in October. Otherwise, biology is unknown.

#### Distribution.

This species is known from a single locality in southeastern Brazil, Santa Catarina: Nova Teutônia.

#### Etymology.

The species is named after Brazil, the country where it was found.

**Figures 68–76.**
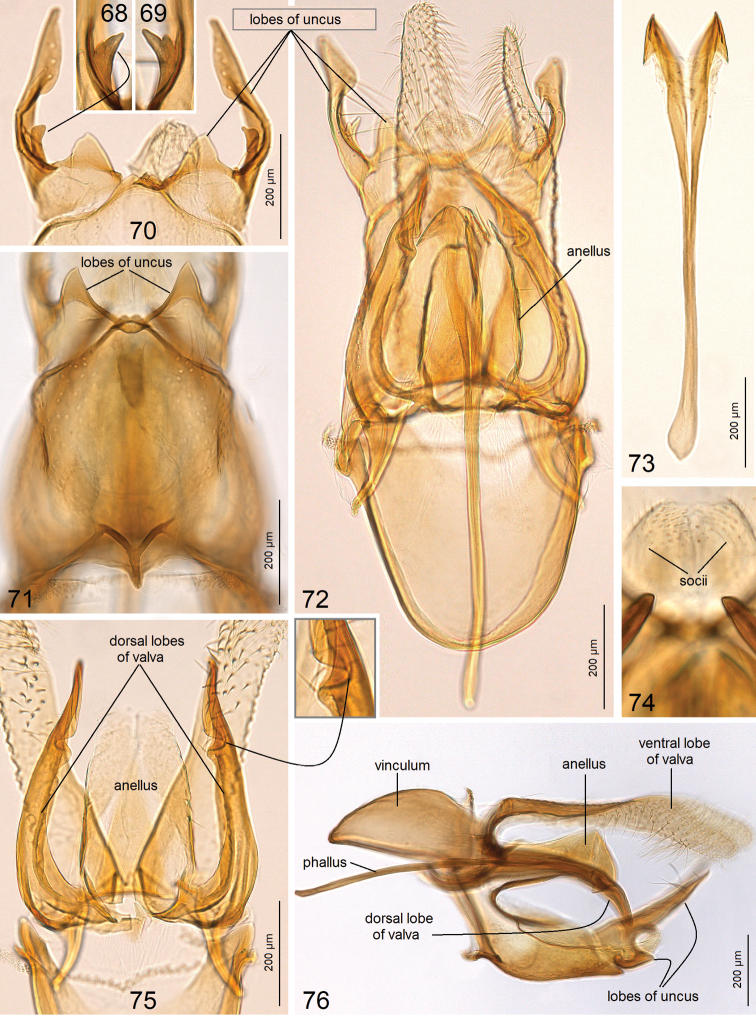
Male genitalia of *Astrotischeria atlantica* sp. nov. **68, 69** inner process of uncus, holotype, genitalia slide no. AD969 **70** uncus, paratype, genitalia slide no. AD970 **71** smaller lobes of uncus and tegumen, holotype, genitalia slide no. AD969 **72** general view of capsule with phallus inside, holotype, genitalia slide no. AD969 **73** phallus, paratype, genitalia slide no. AD970 **74** socii, holotype, genitalia slide no. AD969 **75** dorsal lobes of valvae and anellus, paratype, genitalia slide no. AD970 **76** lateral view of capsule with phallus inside, holotype, genitalia slide no. AD969 (ZIN).

### Extended distribution range of *Paratischeria neotropicana* (Diškus & Stonis, 2015)

The majority of the Neotropical Tischeriidae are known only from their type localities, due to insufficient sampling efforts. *Paratischeria neotropicana* is a leaf miner on *Sida* L., Malvaceae (Fig. 128): larvae produce blotch-like leaf mines (Figs 130–137). In the male genitalia, it is characterized by the unique lobes of the anellus (Fig. 127). Recently this species was recorded as possessing the broadest distribution among the Neotropical Tischeriidae (Diškus and Stonis 2015), with a range from Belize to Peru. However, our study of new, unidentified material expanded the distribution range of this species from Mexico to Bolivia (Fig. 129).

**New material examined.** 2 ♂, 5 ♀: **Mexico**, Veracruz, Boca Del Rio, 19°06'N, 96°06'W (Mexican Field Station), 13–28 Jul1994, reared from *Sida acuta*, *S. rhombifolia*, and *S. spinosa*, P. Juarez, R. Segura and M. Martinez, genitalia slide no. RA1037♂ (USNM); 1 ♂: **Guatemala**, Petén Region, Rio Dulce, 15°38'N, 89°00'W, elevation 300 m, mining larva on *Sida* sp., Malvaceae, 14 Feb 2012, LT-GT Scie. Exp. 2012, genitalia slide no. RA492 (USNM); 18 ♂, 15 ♀: **Belize**, Cayo District, Chiquibul Forest Reserve, Las Cuevas Research Station, 16°43'53"N, 88°59'11"W, 550 m, mining larvae on *Sida* sp., 17 Sep – 17 Nov 1997 and 6 Feb – 13 Jun 1998, O. T. Lewis, genitalia slide nos 010316205♂, 010316206♂, 010316207♂, 010316208♂, 010316209♂, 010316210♂, 010316211♀ (NHMUK); 3 ♂, 5 ♀: **Bolivia**, Nor Yungas Province, Coroico, 16°12'24"S, 67°43'54"W, elevation 1650 m, mining larvae on *Sida* sp., 7–11 Jun 2018, A. Diškus and J. R. Stonis (ZIN).

**Other material examined.** (published by Diškus and Stonis 2015). 4 ♂, 2 ♀, holotype and paratypes: **Peru**, Tambopata Province, Puerto Maldonado, 12°35'33˝S, 69°10'29˝W, elevation 195 m, on *Sida rhombifolia* 16 Oct 2008, A. Diškus, genitalia slide no. AD711♂ (holotype), AD710♂, AD714♀ (paratypes) (ZMUC); 3 ♂, 4 ♀, paratypes: **Ecuador**, Napo Province, SE Tena, Puerto Misahuallí, 1°2'06"S, 77°40'09"W, elevation 400 m, mining larvae on *Sida rhombifolia*, 6 Feb 2007, A. Diškus, genitalia slide no. AD715♂ (ZMUC); 1 ♂ (genitalia from adult in pupal skin, no pinned moth preserved), 4 ♀, paratypes, 1°2'03"S, 77°39'54"W, elevation 395 m, mining larvae on *Sida rhombifolia*, 7 Nov 2007, A. Diškus, genitalia slide nos. AD712♂, AD713♀, AD719♀ (ZMUC); 2 ♂, 1 ♀, paratypes: **Guatemala**, Petén region, El Remate, Tikal, 17°13'22"N, 89°37'24"W, elevation 320 m, mining larvae on *Sida rhombifolia* 06 Feb 2012, LT-GT Scientific Expedition, genitalia slide no. AD716♂ (ZMUC); 1 ♀, paratype: **Belize**, Orange Walk District, Orange Walk, 18°04'40"N, 88°33'28"W, elevation ca. 5 m, mining larva on *Sida rhombifolia*, 9 Feb 2012, LT-GT Scientific Expedition; 2 ♂, 2 ♀, paratypes: Caribbean Archipelago, Ambergris Cay, 17°56'12"N, 87°57'05"W, elevation ca. 5 m, mining larvae on *Sida rhombifolia* 10 Feb 2012, LT-GT Scientific Expedition, genitalia slide no. AD718♂ (ZMUC).

## Discussion

The word “exotic” in this article’s title was borrowed from Edward Meyrick (1854–1938), who discovered and described the record number of the new Microlepidoptera taxa and laid the foundations of the modern systematics of the smallest Lepidoptera, or the so-called Microlepidoptera (Hill 1939, Clarke 1955, Robinson 1986). Obviously, there is no professional in the field of Microlepidoptera taxonomy who would not know the volumes “Exotic Microlepidoptera” by Edward Meyrick (Meyrick 1915a, 1915b, 1921, 1934, 1936); some of these volumes also include descriptions of new Tischeriidae (Meyrick 1915b, 1934, 1936).

Our article deals with distinctive new species exhibiting unusual, “exotic” morphology and provides new host plants not known outside of the Neotropics. The smallest Lepidoptera in the tropics and subtropics are still wrapped in mystery: they have been poorly investigated, are not well known, and the variety of their morphological and ecological adaptations is surprising.

### Novel, atypical morphological characters

Usually, in male genitalia of Tisheriidae, the valva is covered with simple, slender chaetae, only occasionally it bears a pectinifer (Stonis et al. 2020a). In *Paratischeria mesoamericana* sp. nov. we found that the valva is covered with unique, thickened and distally bifid chaetae (Fig. 101).

The female ovipositor of Tischeriidae is not of the piercing type. Females are characterized by two distinct, rounded ovipositor lobes, and only in a few Malvaceae-feeding *Astrotischeria* species these ovipositor lobes are greatly or fully reduced (Stonis et al. 2019b, 2020a). In the course of our study, we found that in *A. atlantica* sp. nov. the ovipositor lobes are modified into an extended, plate-like process, which slightly resembles a piercing ovipositor of some other moth families (Fig. 78). Such a specialized ovipositor or distally bifid chaetae in the male genitalia were not previously known in the Tischeriidae. Moreover, females of *A. atlantica* possess no peg-like setae, but these modified setae are among the most distinct apomorphies and diagnostic characters of the family. Males of *Astrotischeria* species usually possess two pairs of uncus lobes. In *A. atlantica*, the ventral lobe of the uncus has a small additional spine-like lobe; such a derived uncus was discovered in Tischeriidae for the first time. Moreover, usually in *Astrotischeria* dorsal lobes are long and slender, while ventral lobes are short and rounded. In *A. atlantica* it is an opposite case: ventral lobe greatly developed, while the dorsal lobe, in contrast to other congeneric species, is small and triangular (see Fig. 76).

**Figures 77–80.**
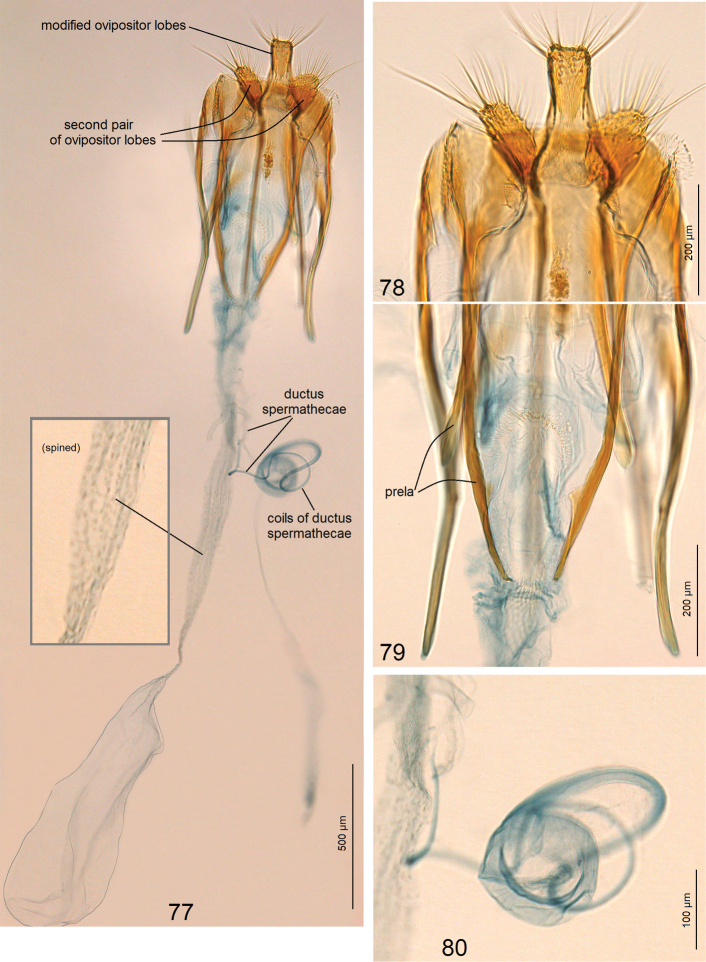
Female genitalia of *Astrotischeria atlantica* sp. nov. **77** paratype, genitalia slide no. AD968, general view **78** same, ovipositor lobes **79** same, apophyses and prela **80** same, coils of ductus spermathecae (ZIN).

Xu et al. (2017) postulated that many large coils of ductus spermathecae in the female genitalia are characteristic exclusively for *Paratischeria* Diškus & Stonis, and it was expected that species of *Astrotischeria* Puplesis & Diškus would possess only a few, small coils. During our study we discovered that, in contrast to other *Astrotischeria*, *A. jociui* sp. nov. possesses many large coils in the ductus spermathecae (Fig. 66). On the other hand, we discovered that females of *A. cornuata* sp. nov. possess a sinuous ductus spermathecae (Fig. 89), but have no distinct coils at all. It is the first species of *Astrotischeria* to be discovered without coils in the ductus spermathecae. Moreover, a strong reduction of the ovipositor lobes is among the most distinct apomorphies and diagnostic characters of the genus; however, we found that in females of *A. cornuata*, the ovipositor lobes are greatly developed, i.e., very large and rounded (Fig. 90).

**Figures 81–88.**
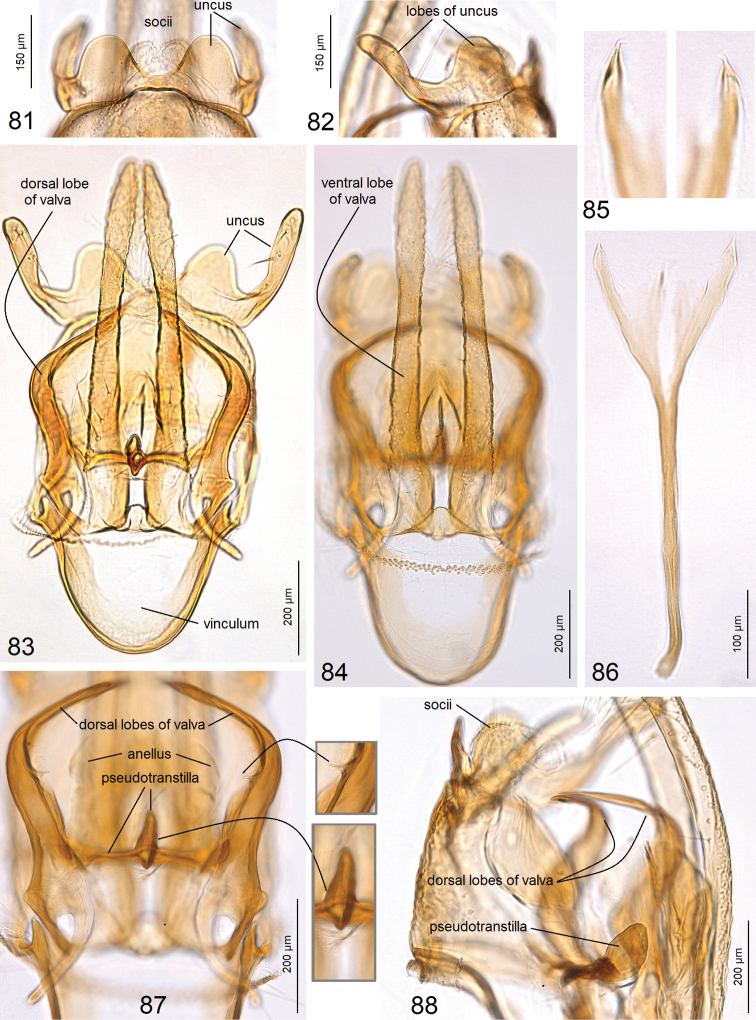
Male genitalia of *Astrotischeria cornuata* sp. nov. **81** uncus, paratype, genitalia slide no. AD975, ventral view **82** same, lateral view **83** general view of capsule with phallus removed, holotype, genitalia slide no. AD522 **84** same, focused on valvae, paratype, genitalia slide no. AD975 **85** apex of phallus, paratype, genitalia slide no. AD975 **86** general view of phallus, holotype, genitalia slide no. AD522 **87** dorsal processes of valvae and pseudotranstilla, paratype, genitalia slide no. AD975, ventral view **88** same, lateral view (ZIN).

Previously, within Tischeriidae only species of *Coptotriche* Walsingham were known to possess a transtilla in the male genitalia. The transverse bar that we discovered between the valvae in *A. cornuata* sp. nov. does not seem to be homologous to the transtilla in *Coptotriche*, because in *A. cornuata* it is not attached to the base of the basal process of the valva, and it represents a novel character for *Astrotischeria*; we propose to use the term pseudotranstilla for this structure (Fig. 87).

Usually Tischeriidae species can hardly be differentiated externally from each other because of their simple and very similar forewing pattern. However, the discovered *Paratischeria suprafasciata* sp. nov. possesses a unique, very distinctive forewing pattern (see Fig. 47).

*Paratischeria braziliensis* sp. nov. represents the most bizarre species in the genus: so far there is no known species with a greatly extended, rod-like vinculum and spiny phallus. These characters are novel to *Paratischeria*, and they resemble, but are probably not homologous, to the characters in *Coptotriche*. Moreover, *P. braziliensis* does not have a transtilla that is so characteristic for *Coptotriche*.

**Figures 89–91.**
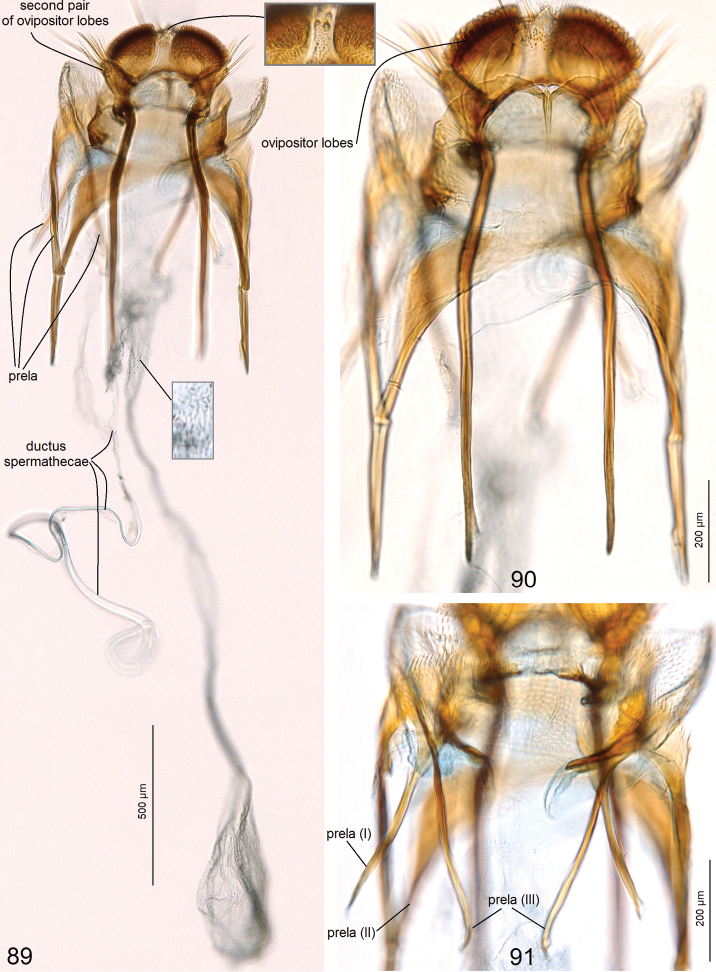
Female genitalia of *Astrotischeria cornuata* sp. nov. **89** paratype, genitalia slide no. AD981, general view **90** same, ovipositor lobes and apophyses **91** same, prela (ZIN).

### Trophic relationships of global and Neotropical Tischeriidae

Tischeriidae are trophically associated with plants belonging to rosid and asterid I core eudicot angiosperms. Recently, the following seventeen host-plant families were known to be hosts for the Tischeriidae family worldwide: Euphorbiaceae, Hypericaceae (Malpighiales), Fabaceae (Fabales), Rhamnaceae, Rosaceae, Ulmaceae, Urticaceae (Rosales), Betulaceae, Fagaceae (Fagales), Combretaceae (Myrtales), Anacardiaceae (Sapindales), Malvaceae, including the former families Sterculiaceae and Tiliaceae (Malvales), Ericaceae, Theaceae, Symplocaceae (Ericales), Apocynaceae (Gentianales), and Asteraceae (Asterales) (Stonis et al. 2017, Xu et al. 2018). Now, because of our discovery of *P. suprafasciata* sp. nov. feeding on *Allophyllus edulis*, we added one more host-plant family to the list, Sapindaceae (Sapindales).

**Figures 92–97.**
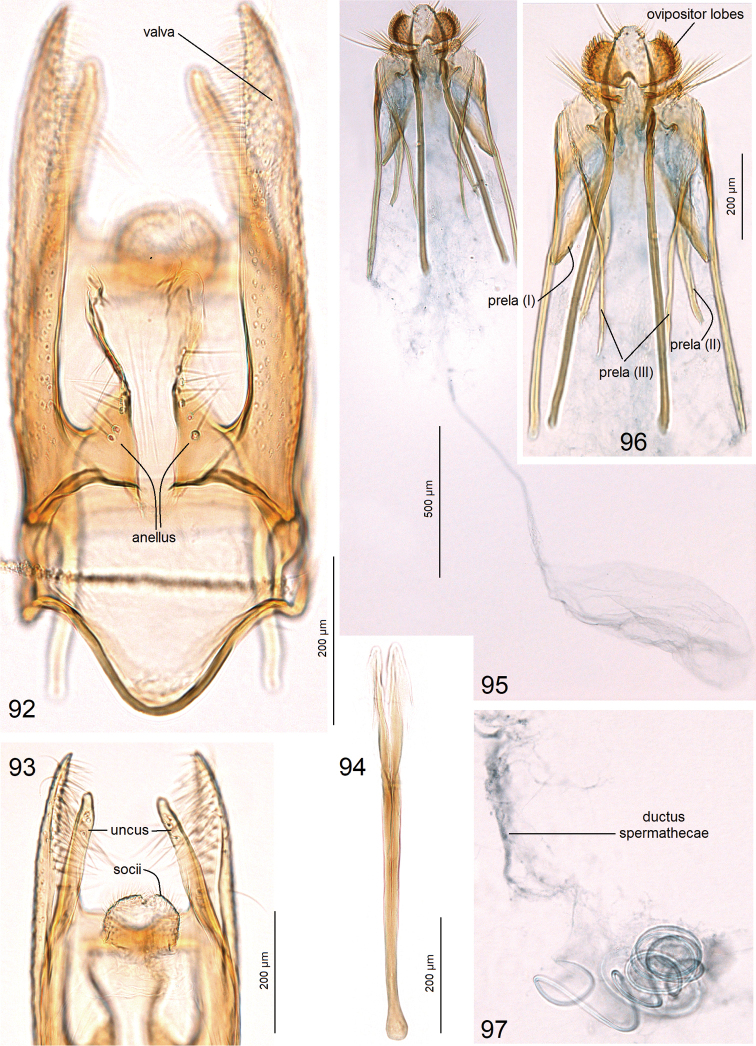
Genitalia of *Paratischeria guarani* sp. nov. **92** holotype, slide no. AD988, male genitalia, general view of capsule with phallus removed **93** same, uncus and socii **94** same, phallus **95** paratype, slide no. AD987, female genitalia, general view **96** same, ovipositor lobes and apophyses **97** same, coils of ductus spermathecae (ZIN).

In the Neotropics, Tischeriidae have been recorded feeding on seven host-plant families: Rhamnaceae, Urticaceae (Rosales), Combretaceae (Myrtales), Sapindaceae (Sapindales), Malvaceae (Malvales), Apocynaceae (Gentianales), and Asteraceae (Asterales). Although we discovered Sapindaceae as a new host-plant family, it is only represented by one tischeriid species, so Asteraceae is still the most utilized host-plant family of the Neotropical Tischeriidae.

It is not known why Tischeriidae have been so successful utilizing Asteraceae in the Americas. However, the estimation of Asteraceae richness and taxonomic diversity by Katinas et al. (2007) indicates that Central and South America are characterized by the globally highest number of genera. It should be also mentioned that the earliest fossils confidently assigned to Asteraceae suggest a South American-Antarctica origin (Barreda et al. 2010, 2012). Recent studies showed that Asteraceae is also an important host for some other leaf-mining families in South America, notably the Nepticulidae (Stonis et al. 2018b) and Bucculatricidae (Vargas et al. 2012). It is interesting to note, that no Asteraceae-feeding Tischeriidae are known outside the Americas. Similar situation was observed with the Asteraceae-feeding Nepticulidae (for a review see Stonis et al. 2018b). Outside of the Americas, Asteraceae-feeding Nepticulidae were found only in North Africa (a single species) and New Zealand where Nepticulidae fauna is dominated by Asteraceae-feeders and the proportion of Asteraceae miners is at least 54% or higher (R. Hoare, pers. comm.).

Below, for the first time, we provide a full list of Tischeriidae host plants from the Neotropics (Table 1).

**Figures 98–104.**
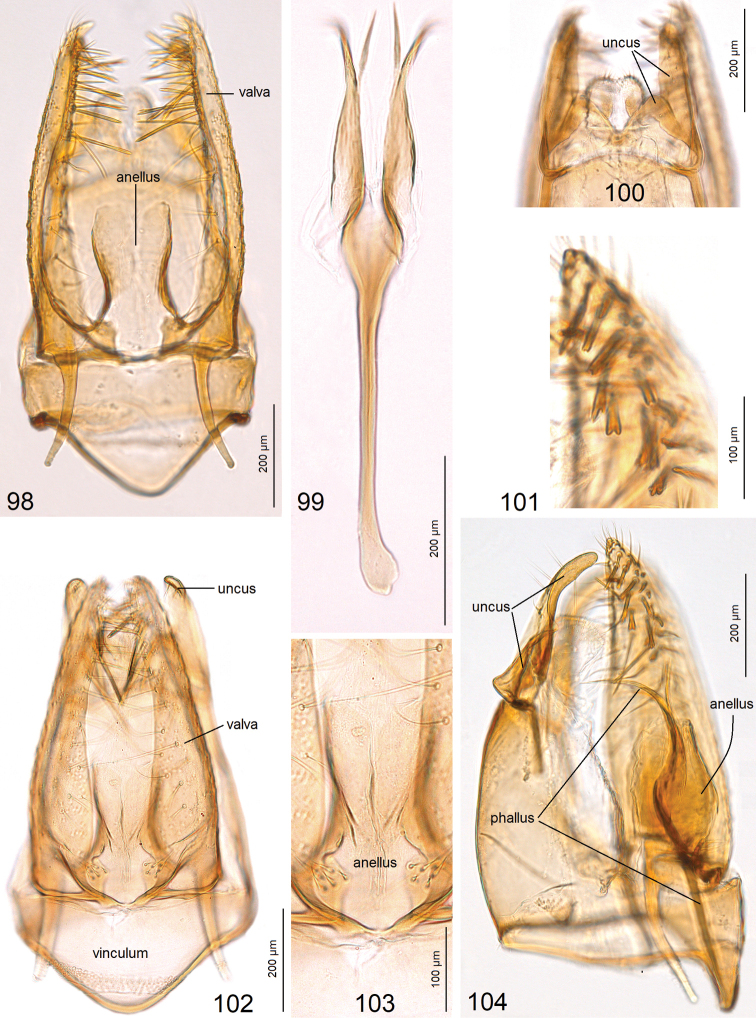
Male genitalia of *Paratischeria mesoamericana* sp. nov. **98** holotype, slide no. AD1005, general view of capsule with phallus removed **99** same, phallus **100** same, uncus and socii **101** same, thickened bifid chaetae of valva **102, 103** capsule with phallus removed, ventral view, paratype, genitalia slide no. AD871 **104** same, lateral view with phallus inside, holotype, genitalia slide no. AD1005 (ZIN).

**Figures 105–110.**
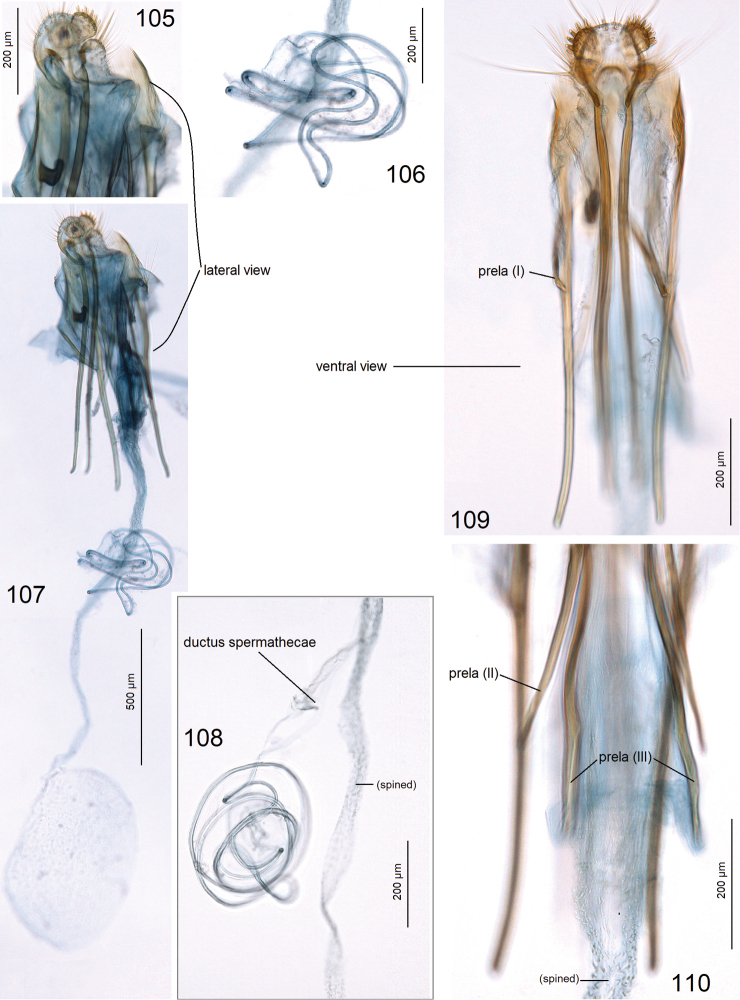
Female genitalia of *Paratischeria mesoamericana* sp. nov. **105** paratype, genitalia slide no. AD1006, ovipositor, lateral view **106** same, coils of ductus spermathecae **107** same, general view **108** same, coils of ductus spermathecae **109** same, ovipositor lobes and apophyses **110** same, prela (ZIN).

**Table 1.** List of currently known host plants of the Neotropical Tischeriidae.

**Rhamnaceae**:	
*Gouania polygama* (Jacq.) Urb.	*Tischeria gouaniae* (Stonis & Diškus, 2007)
**Sapindaceae**:	
*Allophylus edulis* (A. St.-Hil., A. Juss. & Cambess.) Hieron. ex Niederl.	*Paratischeria suprafasciata* sp. nov. (new record)
**Malvaceae**:	
*Sida glabra* Mill.	*Astrotischeria* sp. (Stonis et al. 2020a)
*S. rhombifolia* L., *S. spinosa* L., *S. acuta* Burm.f.	*Paratischeria neotropicana* (Diškus & Stonis, 2015)
*Wissadula* sp., possibly *W. amplissima* (L.) R. E. Fr.	*Astrotischeria ochrimaculosa* Diškus, Stonis & Vargas (Stonis et al. 2019b)
*W. excelsior* (Cav.) C. Presl.	*Astrotischeria jociui* sp. nov. (new record)
**Combretaceae**:	
*Terminalia australis* Cambess.	*Coptotriche parvisacculata* Diškus & Stonis (Stonis et al. 2019c)
**Apocynaceae**:	
*Forsteronia myriantha* Donn. Sm.	*Coptotriche forsteroniae* Stonis & Diškus, 2008
**Asteraceae**:	
*Austroeupatorium inulifolium* (Kunth) R. M. King & H. Rob.	*Astrotischeria trilobata* Diškus & Stonis (Stonis et al. 2018a)
*Baccharis emarginata* (Ruiz & Pav.) Pers.	*Astrotischeria bacchariphaga* Diškus & Stonis (Stonis et al. 2019c)
*B. latifolia* (Ruiz & Pav.) Pers.	*Astrotischeria bacchariphaga* Diškus & Stonis (Stonis et al. 2019c)
*B. spicata* (Lam.) Baill.	*Astrotischeria atlantica* sp. nov. (new record)
*Elephantopus mollis* Kunth	*Paratischeria guarani* sp. nov. (new record)
*Eupatorium* sp.	*Astrotischeria truncata* Diškus & Stonis (Stonis et al. 2019c)
*Lasianthaea fruticosa* (L.) K. M. Becker	*Astrotischeria* spp. (Stonis et al. 2020a)
*Montanoa atriplicifolia* (Pers.) Sch. Bip.	*Astrotischeria casila* Diškus & Stonis (Stonis et al. 2018a)
*M. hibiscifolia* Benth.	*Paratischeria mesoamericana* Diškus & Stonis (new record)
*Otopappus verbesinoides* Benth.	*Paratischeria* sp. (Stonis et al. 2020a)
*Podanthus ovatifolius* Lag.	*Astrotischeria chilei* Puplesis & Diškus (Puplesis and Diškus 2003)
*Rhysolepis incana* (Pers.) H. Rob. & A. J. Moore	*Astrotischeria plagifera* (Meyrick) (Stonis et al. 2018a)
*Scalesia affinis* Hook. f.	*Astrotischeria alcedoensis* Landry (Landry and Roque-Albelo 2004)
*S. baurii* B.L. Rob. & Greenm.	*Astrotischeria scalesiaella* Landry (Landry and Roque-Albelo 2004)
*S. pedunculata* Hook. f.	*Astrotischeria scalesiaella* Landry (Landry and Roque-Albelo 2004)
*Synedrella nodiflora* (L.) Gaertn.	*Astrotischeria selvica* Diškus, Carvalho-Filho & Stonis (Stonis et al. 2018a)
*Sphagneticola trilobata* (L.) Pruski	*Astrotischeria selvica* Diškus, Carvalho-Filho & Stonis (Stonis et al. 2018a)
*Tessaria integrifolia* Ruiz & Pav.	*Astrotischeria koehleri* (Bourquin 1962)
*Tilesia baccata* (L.) Pruski	*Astrotischeria selvica* Diškus, Carvalho-Filho & Stonis (Stonis et al. 2018a)
*Wedelia calycina* Rich.	*Astrotischeria colombiana* Stonis & Vargas (Stonis et al. 2019b)
**Urticaceae**:	
*Phenax hirtus* (Sw.) Wedd.	*Paratischeria ferruginea* Diškus & Stonis (Stonis et al. 2017)

### Predicting distribution through host-plant distribution of the host-specific Tischeriidae

Currently, only three Tischeriidae species have been recorded over a broad range in the Neotropics: *Paratischeria neotropicana* (Diškus & Stonis), occurring from Mexico to Bolivia (Fig. 129), *Astrotischeria selvica* Diškus, Carvalho-Filho & Stonis, occurring from Central America to the Atlantic coast of equatorial Brazil (Stonis et al. 2018a), and *A. ochrimaculosa* distributed from Colombia to Peru (Stonis et al. 2019b). All remaining Neotropical species are known from a single locality, or restricted area, which suggests that they are poorly sampled. On the other hand, leaf-mining larvae of Tischeriidae show a great selectivity in their food choice: they are stenophagous (monophagous or oligophagous). Therefore, despite the fact that insect distribution may depend on many more factors than host plant alone, recently we hypothesized that the distribution of the host plants can suggest much broader ranges for these host-specific leaf miners (Stonis et al. 2019c). We discuss potential distribution ranges of the species described in this paper.

**Figures 111–115.**
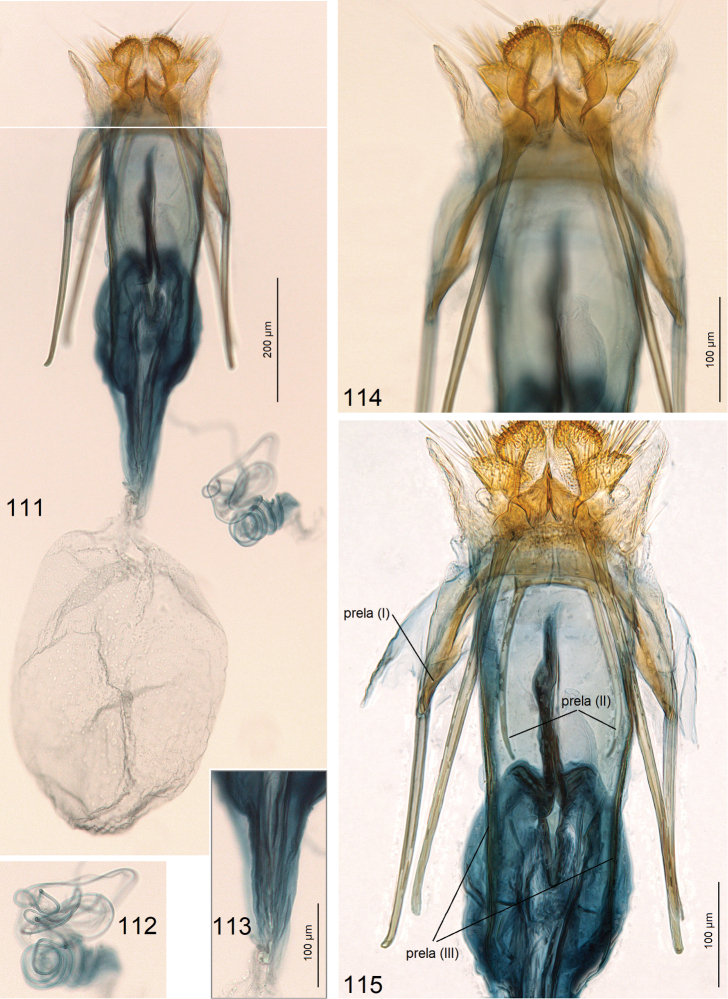
Female genitalia of *Paratischeria suprafasciata* sp. nov. **111** holotype, genitalia slide no. AD967, general view **112** same, coils of ductus spermathecae **113** same, fragment of corpus bursae **114** same, ovipositor lobes **115** same, apophyses and prela (ZIN).

**Figures 116–126.**
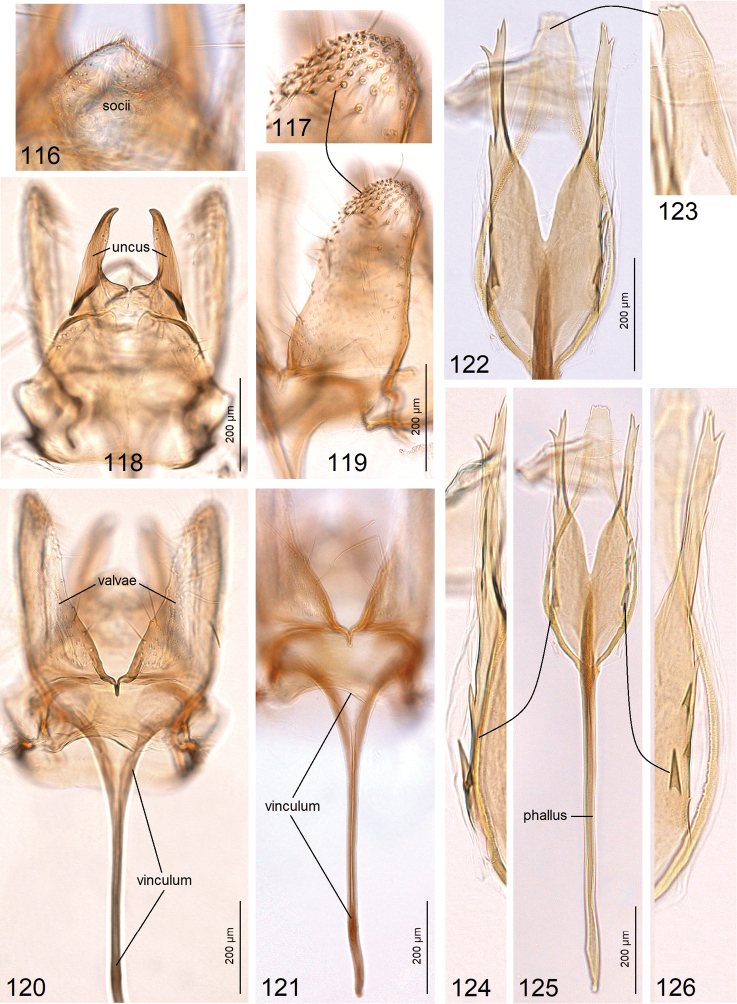
Male genitalia of *Paratischeria braziliensis* sp. nov. **116** holotype, slide no. AD1004, socii **117** same, apex of valva **118** same, uncus and tegumen **119** same, valva **120, 121** same, vinculum **122–126** same, phallus (USNM).

*Astrotischeria jociui* sp. nov. is currently known only from a single locality in Peru, and feeds on *Wissadula* Medik., Malvaceae. It is currently estimated that *Wissadula* consists of approximately 32 species. The largest number of species occurs in the Neotropics, with the highest concentration in southeastern Paraguay, northern Argentina, and midwestern Brazil (Fig. 138), and with a few species in North America, Asia, and Africa (Bovini and Baumgratz 2016). We expect this tischeriid species may also occur in other countries in South America and possibly in Central America, and in sunny areas with disturbed vegetation, rarely in forests.

*Astrotischeria atlantica* sp. nov. is currently known only from a single locality in Uruguay, Rocha Department, La Paloma, and feeds on *Baccharis spicata* (Lam.) Baill., Asteraceae. *Baccharis spicata*, “chilca blanca” or “chilca amarga”, is a dioecious, rhizomatous shrub or subshrub native to Bolivia, Paraguay, southern Brazil, Uruguay, and central and northeastern Argentina (Fig. 139); recently it has been reported as an invasive in Europe (Verloove et al. 2017). We expect that this species also occurs in other South American countries, and mainly in grasslands and roadsides.

**Figures 127–137.**
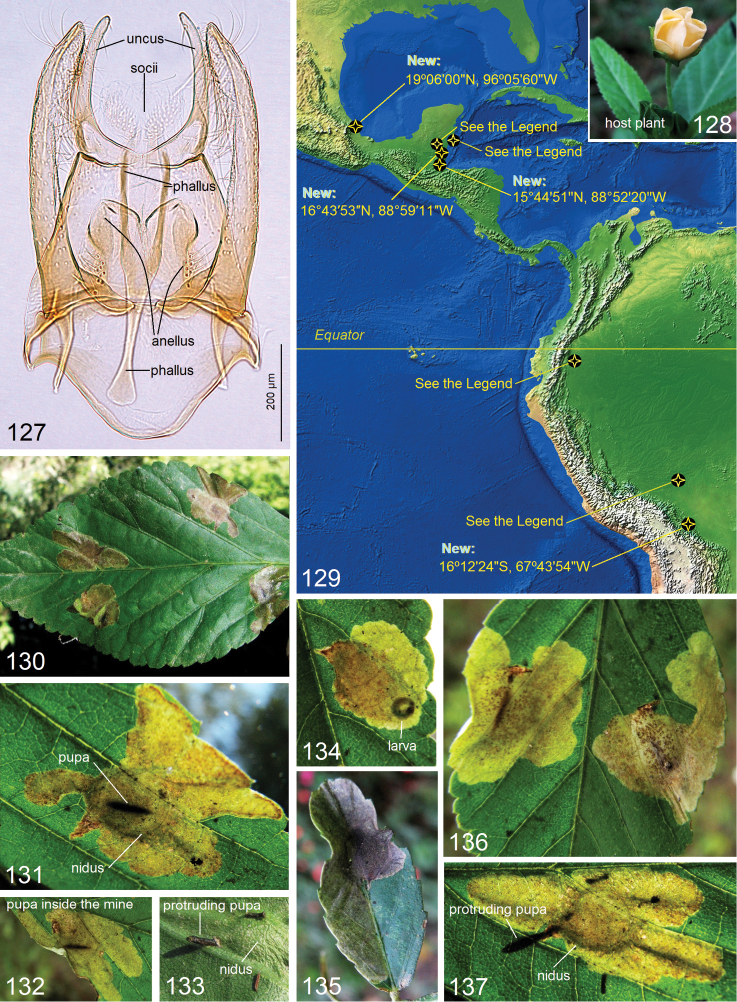
*Paratischeria neotropicana* (Diškus & Stonis, 2015). **127** male genitalia, capsule with phallus inside, Rio Dulce, Guatemala, genitalia slide no. RA492 (USNM) **128** host plant *Sida* L., Malvaceae**129** distribution map (for the localities see Material examined) **130–137** leaf mines, Coroico, Bolivia.

*Paratischeria guarani* sp. nov. is currently known only from a single locality in Paraguay, Departamento de Itapúa, Hohenau and feeds on *Elephantopus mollis* Kunth, Asteraceae. *E. mollis*, or “elephant’s foot”, is an herbaceous perennial plant with compound flower heads, native to the American tropics and subtropics (Dematteis 2014). It is an invasive weed and it has been widely introduced elsewhere (e.g., Africa, Asia, Australia, and the Pacific) (CABI 2020). In the Americas, we expect this tischeriid species may occur from Mexico and the Caribbean to Argentina (Fig. 140), in high rainfall areas with fertile tropical conditions, especially in open areas, pastures, plantations, forest edges, roadsides, and disturbed or marshy areas.

*Paratischeria mesoamericana* sp. nov. is currently known only from a single locality in Guatemala, Antigua Guatemala, San Juan del Obispo, and feeds on *Montanoa hibiscifolia* Benth., Asteraceae. *M. hibiscifolia*, known in Guatemala as “cajete”, “cana rancho”, “quil”, “toquillo”, “vara de jaula”, “xixil” (Tropicos 2020), is a southern North American and Central American shrub with 3–5-lobed leaves and prominent petiolar auricles (Funk 1982). We expect this tischeriid species to also occur in Costa Rica, Nicaragua, Belize, and north to Chiapas in Mexico, in pine-oak forests, on hillsides and along streams, lakes and roads from 350 to 2500 m.

**Figures 138–141.**
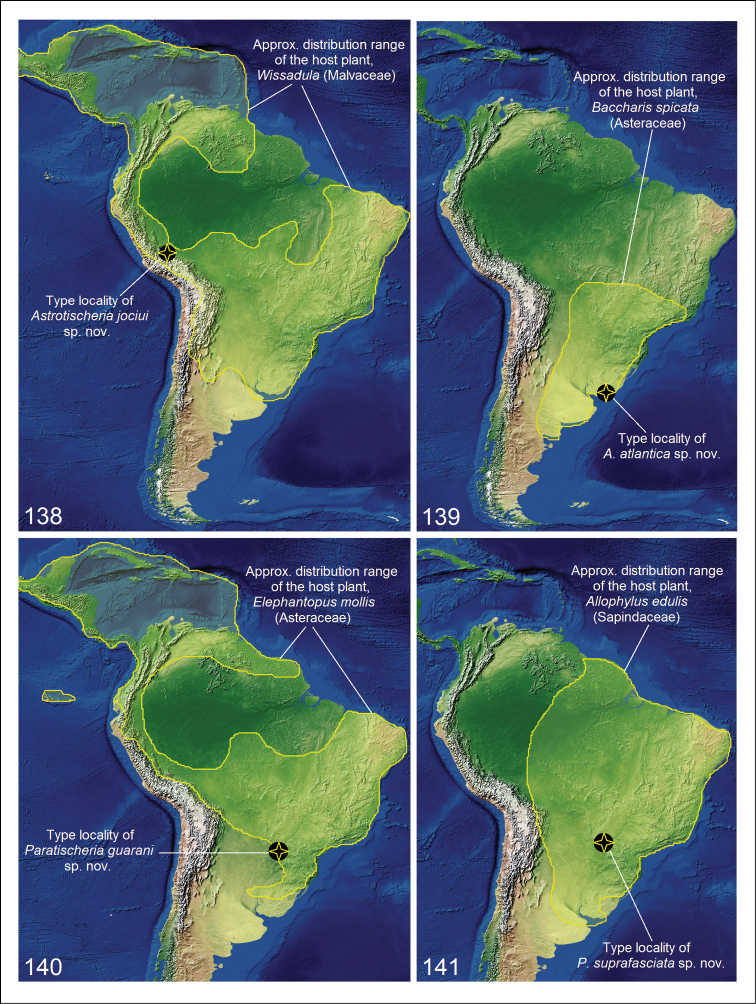
Predicted host-specific Tischeriidae distribution from host plant distribution. **138***Astrotischeria jociui* sp. nov. **139***A. atlantica* sp. nov. **140***Paratischeria guarani* sp. nov. **141***P. suprafasciata* sp. nov.

*Paratischeria suprafasciata* sp. nov. is currently known only from a single locality, Puerto Iguazú, in northeastern Argentina, and feeds on *Allophylus edulis* (A. St.-Hil., A. Juss. & Cambess.) Hieron. ex Niederl., Sapindaceae. *A. edulis*, the “cocú” or “chal chal”, is a South American shrub or little tree with persistent, 3-foliate leaves and edible, red fruits, extending from Guiana to Argentina (Ferrucci 2004) (Fig. 141). We expect this tischeriid species to occur in the tropical, subtropical, and riverine forests of Guianas, Brazil, Paraguay, Uruguay, and north to central Argentina.

### Summary on species description of the Neotropical Tischeriidae

**Figures 142–144.**
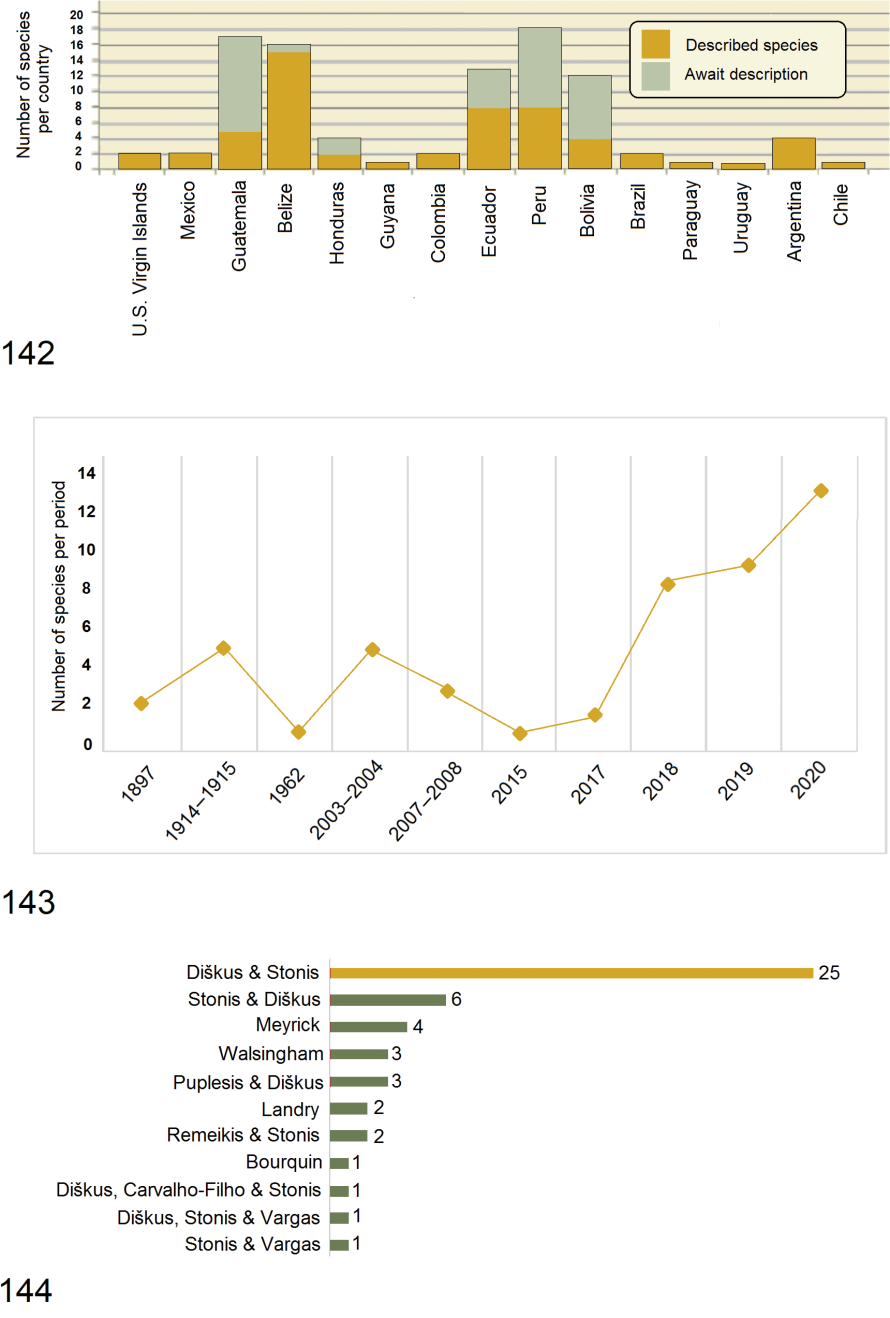
Overview on the Neotropical Tischeriidae fauna and history of the description of species. **142** currently described diversity of Tischeriidae per country (Note that some species occur in more than one country, therefore, there is some overlap so the total in the graph does not agree with the total 49 species known from the Neotropics) **143** description history of the Tischeriidae from the Neotropics **144** authorship of all currently known Tischeriidae species of the fauna of the Neotropics.

The study of the Tischeriidae fauna in the Neotropics began in the late nineteenth to early twentieth centuries, but only during the last decade the inventory and especially collecting of the Neotropical Tischeriidae has become more purposeful and active (Fig. 143). The overall impression is that the Neotropical fauna is an isolated entity: there is no overlap at the species level between the fauna of the Neotropics and that of the rest of world, including the adjacent Nearctic region. The total number of Tischeriidae of the Neotropics now numbers 49 described species including the seven new species described in this paper and six new species by Stonis et al. 2020a. Additionally, some other new species have already been recognized, dissected, and are under preparation for publication by us (see Fig. 142). The number of described Tischeriidae species by country is unequal, mostly due to different research effort (Fig. 142). Some species occur in more than one country, therefore, there is some overlap, and the total (57) does not agree with the total of 49 species known from the Neotropics.

In total, the world fauna of Tischeriidae now numbers 158 described species, but only 153 species are named. Five South African species were documented and published but were left unnamed because of lack of males (Puplesis and Diškus 2003, Stonis et al. 2019a). Thus, the Neotropical fauna forms one third of the currently known global fauna. The history of species description is given in Fig. 143, the authorship in Fig. 144. In total, eight researchers were involved in species descriptions from the Neotropics, some of them described at least one or two species of Tischeriidae, others are responsible for the bulk, Notably Arūnas Diškus who, in the last two decades, is responsible for the discovery and descriptions (all with co-authors) of 36 species.

## Supplementary Material

XML Treatment for Astrotischeria jociui

XML Treatment for Astrotischeria atlantica

XML Treatment for Astrotischeria cornuata

XML Treatment for Paratischeria guarani

XML Treatment for Paratischeria mesoamericana

XML Treatment for Paratischeria suprafasciata

XML Treatment for Paratischeria braziliensis
